# Supplementary material for: Improvement of SMN2 Pre-mRNA Processing Mediated by Exon-Specific U1 Small Nuclear RNA
Source: Am J Hum Genet. 2015 Jan 8;96(1):93–103. doi: 10.1016/j.ajhg.2014.12.009 (PMC4289686; doi:10.1016/j.ajhg.2014.12.009)
Supplement: Document S1. Figures S1–S6 and Table S1 [file mmc1.pdf]

The American Journal of Human Genetics

Supplemental Data

Improvement of SMN2 Pre-mRNA Processing Mediated by Exon-Specific U1 Small Nuclear RNA

Andrea Dal Mas, Malgorzata Ewa Rogalska, Erica Bussani, and Franco Pagani

Supplementary Material

S1

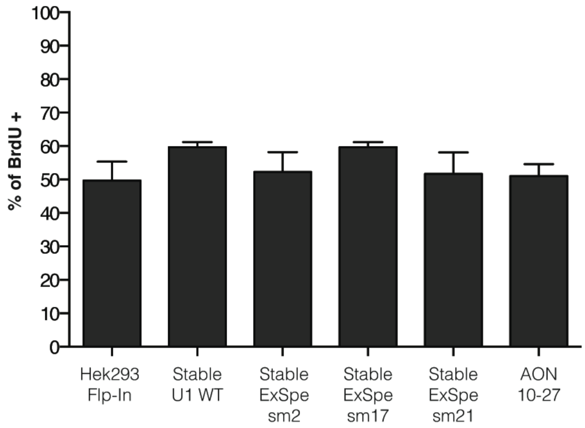

Figure S1.

Proliferation assay for Hek293 Flp-In cells, ExSpe U1 or U1 WT stable clones. BrdU staining of cells expressing one additional copy of U1 snRNA (U1 WT) gene ore one copy of ExSpe U1 sm2, sm 17 or sm21 genes. The histograms represent the percentage of BrdU positive cells (BrdU+) and are the mean +/- SD of three independent experiments.

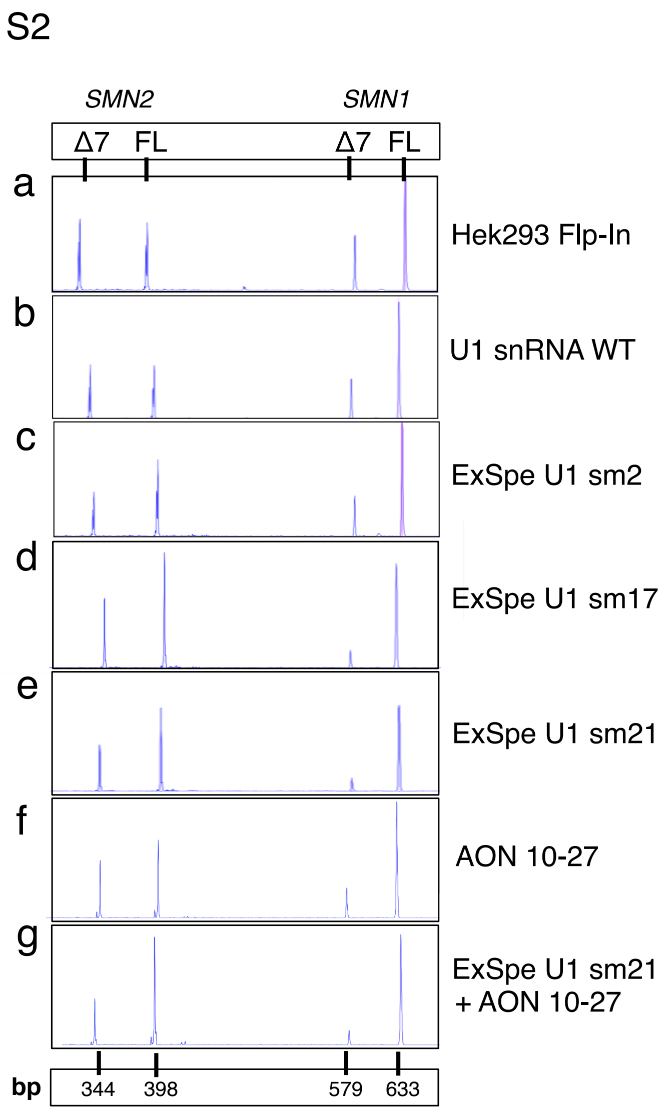

Figure S2.

Capillary electrophoresis output of SMN1 and SMN2 transcripts in Hek293 Flp-In cells. Total RNA was extracted from Hek293 Flp-In cells stably expressing one copy of U1 WT or ExSpe sm2, sm17 or sm21 and cells transfected with antisense oligonucleotide 10-27 (AON 10-27) and ExSpe sm21 stable clone transfected with AON 10-27. RT-PCR was performed with FAM-labelled-E6-Fw and E8-467-Rev primers. The corresponding amplified products were digested with DdeI restriction enzyme in order to discriminate fragments coming from *SMN1* or *SMN2.* The resulting products were run on capillary electrophoresis. Each peak reported in the graph represent an isoform and the area surrounding the peaks gives the relative quantification of the corresponding isoforms. The identity of each peak (FL containing exon 7 or ΔE7 with exon 7 skipped) and its provenience (*SMN1 or SMN2*) is indicated on the top of the panel. The size is indicated below.

S3

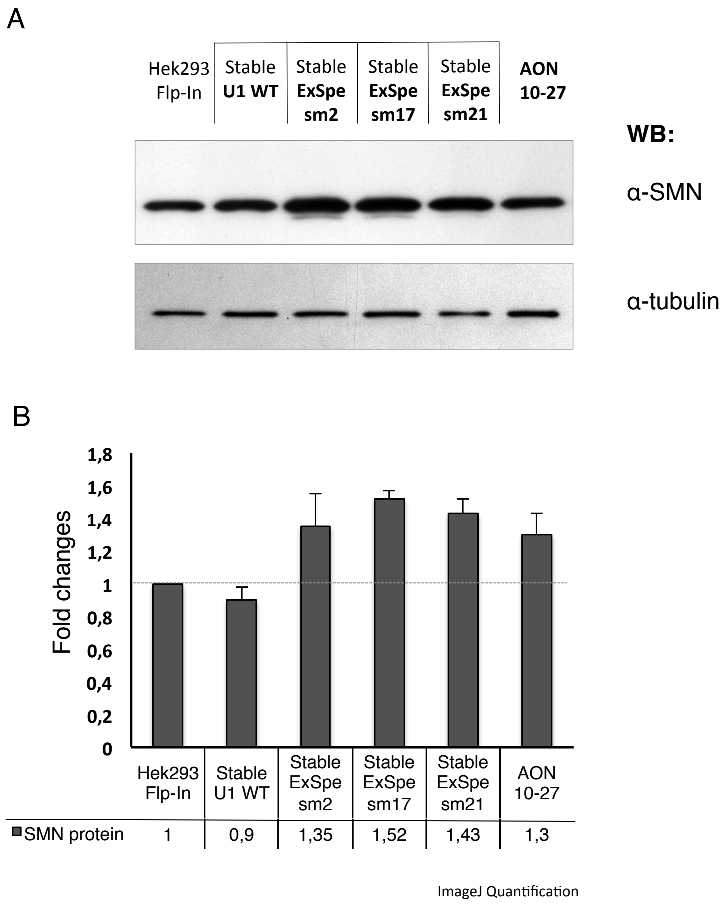

Figure S3

Expression of ExSpe U1 from a single chromosome-integrated copy of the gene increases the amount of SMN protein in Hek293 Flp-In cells.

(a) Protein extract from Hek293 Flp-In stable clones expressing one copy of ExSpe U1 sm2, sm17, sm21 or U1 WT were loaded on 4-12% SDS-PAGE. SMN protein was detected by Western blotting and the same blot was re-probed with antibody against tubulin as a protein loading control.

(b) Intensity of protein bands in (a) was estimated through ImageJ-Quantification program. The histograms indicate the SMN protein fold increase relative to the amount of SMN protein in Hek293 Flp-In (value set to 1). Error bars show standard deviation.

S4

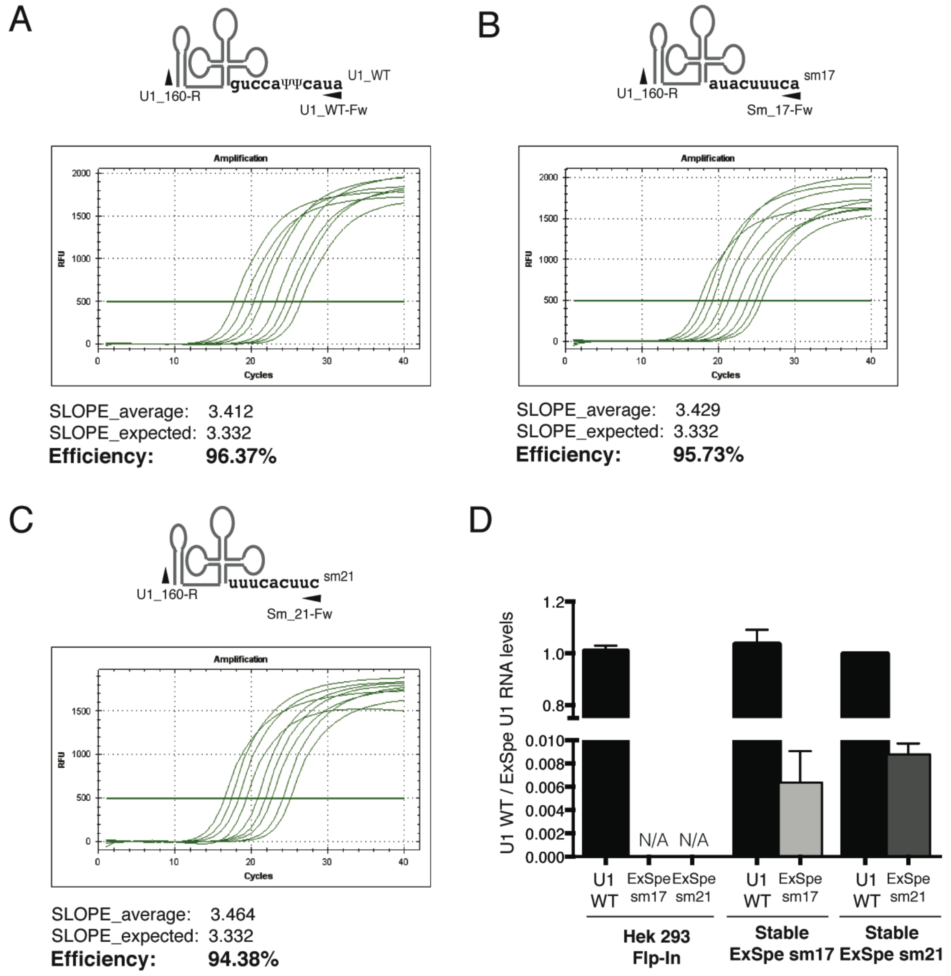

Figure S4.

Analysis of ExSpe U1 sm17 and sm21 expression levels.

(a-b-c) The three RT-PCR reactions used to determine the levels of U1 snRNA WT, ExSpe sm17 and ExSpe sm21 have equivalent efficiencies. ExSpe U1 sm2 was not analysed, as PCR could not discriminate between ExSpe U1 sm2 and U1 snRNA 5'-tails, due to the high sequence similarity. Plasmid DNA dilutions were used as template to determine the efficiencies of PCR reactions. Calibration curves show that the efficiencies are similar, thus allowing to compare them directly and to estimate absolute levels of ExSpe U1s.

(d) Total RNA was extracted from the indicated cells and treated with DNAse. Retrotranscription reaction has been performed +/-RT to check for DNA contamination. Sybr-based qPCR has been performed using specific primers to detect U1 snRNA WT, ExSpe U1 sm17 and ExSpe U1 sm21. The amount of ExSpe U1 for each cell line has been expressed as ratio to endogenous U1 snRNA WT level.

S5

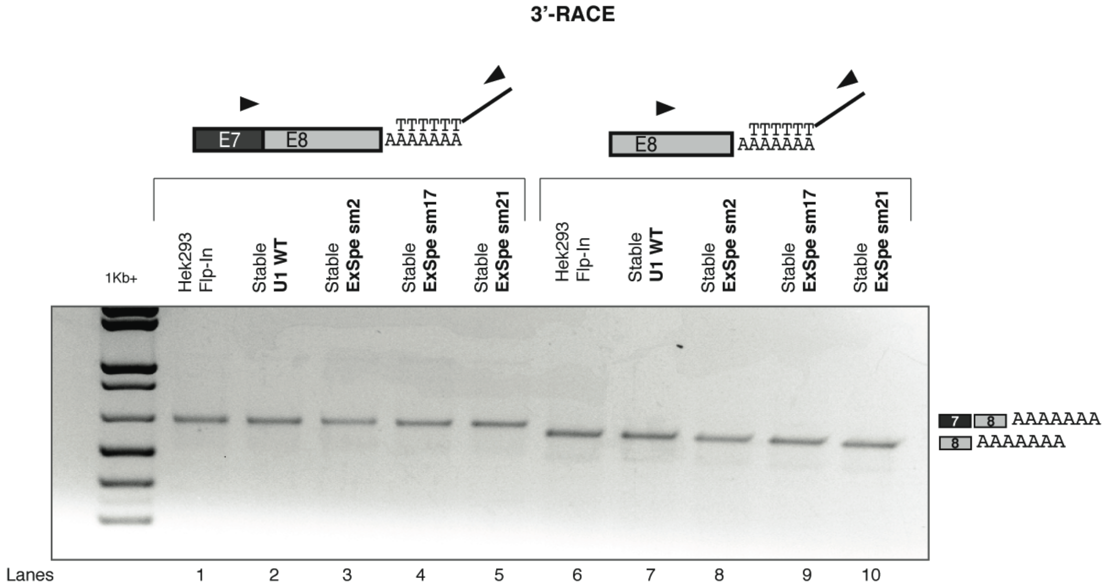

Figure S5.

3'-RACE (Rapid amplification of cDNA ends) assay. Total RNA was extracted from the indicated cells and retrotranscribed using an oligo dT primer with a specific anchor. Polyadenylated transcripts from SMN genes were evaluated using a primer located on exon 7 (lanes 1-5) or on exon 8 (lanes 6-10) in combination with a specific primer located on the anchor. The amplified fragments were separated on 1.5% agarose gel. The identity of the bands was verified through direct sequencing of the PCR products and has been schematically reported on the right of the gel.

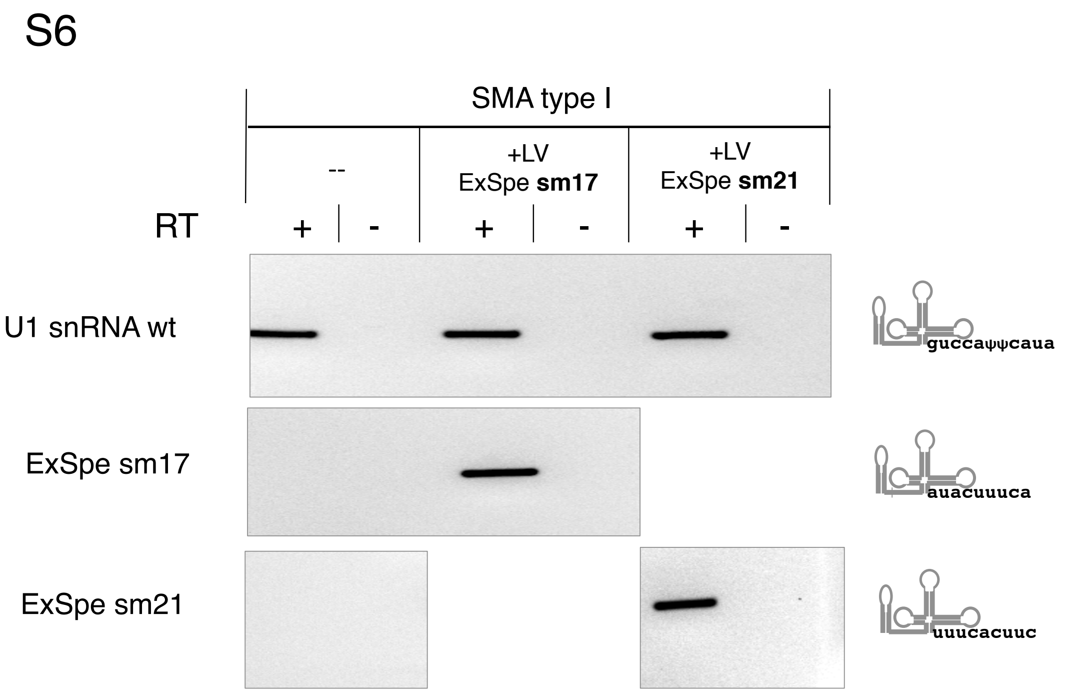

Figure S6.

Expression of Exon-Specific U1 RNAs after lentiviral transduction in SMA type I fibroblasts. Total RNA was extracted from SMA type I fibroblasts treated with lentiviral particle expressing ExSpe U1 sm17 or sm21 and treated with DNAse. RT-PCR has been performed +/- retrotranscriptase (+/-RT) to check for DNA contamination using specific set of primers to amplify the endogenous U1 WT, the ExSpe sm17 or the ExSpe sm21. The primers used for each amplification are indicated on the left of the panel and the identity of the PCR product is schematically reproduced on the right.

Primer name	Sequence (5’- -3’)
E6-Fw_FAM	5’-*FAM*-ATAATTCCCCCACCACCTCCC-3’
E8-467-Rev	5’-TTGCCACATACGCCTCACATAC-3’
U1sm17-Fw	5’-ATAACTTTCATAGCAGGGGGAGAT-3’
U1sm21-Fw	5’-ATAATTCACTTTGCAGGGGGAGAT-3’
U1-160 Rev	5’-GGAAAGCGCGAACGCAGTC-3’
Ex2a Fw	5’-AGCGATGATTCTGACATTTGGGATG-3’
Ex2b Rev	5’-CTGTTGTAAGGAAGCTGCAGTATTCTT-3’
Int1_13850Fw	5’-GATTAAACCTATCTGAACATGAG-3’
Int2a_14006 Rev	5’-GAAATAAGAAAACGACTAAGCAAG-3’
Int6 Fw	5’-CCATATAAGCTATCTATATATAGCTATCT-3’
Int7 Rev	5’-CATTTGTTTTCCACAAACCATAAAGTT -3’
18S Fw	5’-CCTTTAACGAGGATCCATTGGA -3’
18S Rev	5’-CGAGCTTTTTAACTGCAGCAACT-3’
Oligo dT for 3'-RACE	5’-GACCACGCGTATCGATGTCGACTTTTTTTTTTTTTTTTV-3’
Exon-6_Fw	5’-TGCTTTGGGAAGTATGTTAATTTCATG-3’
Exon-7_Fw	5’-GTGCTCACATTCCTTAAATTAAGG-3’
Exon-8_Fw	5’-GGCATAGAGCAGCACTAAATGACACC-3’
Anchor Rev	5’-GACCACGCGTATCGATGTCGAC-3’

Table S1.

Sequences of the DNA oligonucleotides used for RT-PCR experiments.
