# Supplementary material for: Improvement of SMN2 Pre-mRNA Processing Mediated by Exon-Specific U1 Small Nuclear RNA
Source: Am J Hum Genet. 2015 Jan 8;96(1):93–103. doi: 10.1016/j.ajhg.2014.12.009 (PMC4289686; doi:10.1016/j.ajhg.2014.12.009)
Supplement: Document S2. Article plus Supplemental Data [file mmc2.pdf]

Improvement of SMN2 Pre-mRNA Processing Mediated by Exon-Specific U1 Small Nuclear RNA

Andrea Dal Mas,[1] Malgorzata Ewa Rogalska,[1] Erica Bussani,[1] and Franco Pagani[1,*]

Exon-specific U1 snRNAs (ExSpe U1s) are modified U1 snRNAs that interact with intronic sequences downstream of the 5′ splice site (ss) by complementarity. This process restores exon skipping caused by different types of mutation. We have investigated the molecular mechanism and activity of these molecules in spinal muscular atrophy (SMA), a genetic neuromuscular disease where a silent exonic transition on the survival motor neuron 2 (*SMN2*) leads to exon 7 (E7) skipping. By using different cellular models, we show that a single chromosome-integrated copy of *ExSpe U1* induced a significant correction of endogenous *SMN2* E7 splicing and resulted in the restoration of the corresponding SMN protein levels. Interestingly, the analysis of pre-mRNA transcript abundance and decay showed that ExSpe U1s promote E7 inclusion and stabilizes the SMN pre-mRNA intermediate. This selective effect on pre-mRNA stability resulted in higher levels of SMN mRNAs in comparison with those after treatment with an antisense oligonucleotide (AON) that targets corresponding intronic sequences. In mice harboring the *SMN2* transgene, AAV-mediated delivery of *ExSpe U1* increased E7 inclusion in brain, heart, liver, kidney, and skeletal muscle. The positive effect of ExSpe U1s on SMN pre-mRNA processing highlights their therapeutic potential in SMA and in other pathologies caused by exon-skipping mutations.

Introduction

Pre-mRNA splicing is a finely regulated process that requires specific signals on RNA molecules, such as the 5′ and 3′ splice sites (5′ ss and 3′ ss), the branch point sequence (BPS), and additional less-conserved intronic or exonic elements with enhancer or silencer functions (ISE, ESE, ISS, ESS, respectively).[1,2] These elements drive the spliceosome, the macromolecular complex that catalyzes the splicing reaction, in the identification of the correct exon-intron boundaries among the never-used pseudo-splice sites located in the pre-mRNA molecules.[3] The first step of spliceosome assembly involves the binding of the U1 small nuclear ribonucleoparticle (U1 snRNP) to the 5′ ss of an exon through its 9-bp-long 5′ tail.[4] U1 snRNAs have a stable and defined secondary structure that interacts with a set of U1-specific proteins named U1-A, U1-70K, and U1-C as well as with the Smith antigen (Sm) proteins, common to all U-rich snRNAs.[5] These interactions result in the formation of a functional U1 snRNP. Recently, the biological repertoire of the U1 particle has expanded, because its involvement in different cellular processes beyond splicing has been demonstrated.[6] In particular, U1 snRNPs protect transcripts from premature cleavage and polyadenylation at cryptic polyadenylation signals (PASs) in introns and, when positioned on the first exon, promote transcription.[7–9]

A large proportion of disease-causing mutations has been shown to affect the splicing mechanism, mainly causing the skipping of an exon from the final transcript.[10,11] We recently developed an approach to correct exon skipping based on modified U1 snRNAs, named exon-specific U1s (ExSpe U1s).[12] Differently from previously reported modified-U1-based approaches aimed at reinforcing the binding of the U1 particle at suboptimal 5′ ss,[13–16] ExSpe U1s have engineered 5′ tails that direct their loading onto nonconserved intronic regions downstream of the donor site of a specific exon, reducing undesired off-target events. We previously demonstrated that a number of different ExSpe U1s are able to correct aberrant splicing resulting from different types of mutations in minigene models of *Coagulation Factor IX*, *CFTR*, and *SMN2*.[12]

To characterize the activity of ExSpe U1s and to further investigate their mechanism of action in vivo, in the present work we focused on spinal muscular atrophy (SMA [MIM 253300]). SMA is a recessive-autosomal neuromuscular disease affecting α-motoneurons in the anterior horn of the spinal cord. It is a leading genetic cause of infant mortality, with a carrier frequency of ~1:35 and an incidence of ~1:6,000 newborns. SMA clinical symptoms appear in childhood, are of variable severity, and comprise progressive muscular weakness and atrophy that affect mainly proximal muscles.[17] There is currently no treatment for this pathology. SMA is caused by a homozygous loss of function of survival motor neuron (*SMN1* [MIM 600354]), which encodes for SMN, a key protein in the biogenesis of small ribonucleoparticles (snRNPs), which has recently been associated with a complex system of neuronal circuitry[18–20] and has also been linked to transcription, stress response, apoptosis, axonal transport, and cytoskeletal dynamics.[21–23] However, humans possess a *SMN1* paralog, named *SMN2* (MIM 601627), located as well on chromosome 5, but in centromeric position. The coding sequence of the paralog gene is nearly identical to *SMN1*, with the exception of a silent exonic cytosine-to-thymine (C-T) transition at nucleotide 840, matching the position +6 of exon 7 (E7). This substitution disrupts an

[1]Human Molecular Genetics Laboratory, International Centre for Genetic Engineering and Biotechnology (ICGEB), Padriciano 99, 34149 Trieste, Italy
*Correspondence: pagani@icgeb.org
http://dx.doi.org/10.1016/j.ajhg.2014.12.009. ©2015 The Authors
This is an open access article under the CC BY license (http://creativecommons.org/licenses/by/3.0/).

SRSF1-dependent exonic splicing enhancer (ESE)[24–26] and creates an intronic splicing silencer (ISS) recognized by the inhibitory splicing factor hnRNPA1.[27] As a consequence, the majority of *SMN2* mRNAs lack E7 and lead to the formation of a truncated, highly unstable ΔE7 protein, which rapidly undergoes degradation.[28] The small amount of full-length (FL) protein produced by *SMN2* is therefore essential for survival, and the number of copies of *SMN2* inversely correlate with the severity of the pathology.[17] Although SMA is not a splicing disease per se, *SMN2* is an optimum candidate for a splicing therapy, because all SMA-affected individuals retain at least two copies of *SMN2*. Correction of E7 missplicing can be obtained through antisense masking of an hnRNPA1-dependent intronic splicing silencer N1 (ISS-N1) located downstream of the E7 donor splice site via chemically modified antisense oligonucleotides (AONs) or modified U7 snRNAs.[17,29] In addition, a compound has been recently described that selectively enhances SMN transcripts.[30] The ISS-N1 comprises a 15-bp-long intronic sequence located downstream of E7 5′ ss, spanning from position +10 to +24. Mutagenesis and binding experiments showed the presence of two weak cooperative binding sites for the inhibitory splicing factor hnRNPA1 that recognizes the *CAG* and *AAAG* motifs present in the pre-mRNA molecule at positions +11/+13 and +21/+24, respectively.[31] Here we show that three ExSpe U1s previously reported to be active in *SMN2* minigene assays are able to fully reverse E7 aberrant splicing in fibroblasts from individuals with the severe SMA type I. Transduction of primary cells from SMA-affected individuals with lentiviral particles expressing SMN-specific ExSpe U1s restores normal E7 transcript levels, leading to the production of physiological amounts of functional SMN protein. Adeno-associated virus (AAV)-mediated delivery of ExSpe U1 corrects *SMN2* splicing in transgenic mice. Furthermore, we demonstrate that a single chromosome-integrated copy of SMA-specific *ExSpe U1* is sufficient to influence endogenous *SMN1* and *SMN2* mRNAs by positively modulating E7 processing through a molecular mechanism that differs from the one previously reported for antisense oligonucleotide 10–27 (AON 10–27) that masks a pre-mRNA sequence.

Material and Methods

Cell Culture and Lentiviral Production

HEK293 Flp-In cells and SMA fibroblasts (G3813, G3814 Coriell Institute) were grown in Dulbecco's modified Eagle's medium with Glutamax I (GIBCO) (DMEM with glutamine, sodium pyruvate, pyridoxine, and 4.5 g/l glucose) supplemented with 10% fetal calf serum (Euro Clone) and antimycotic (Sigma) according to the manufacturer's instruction.

Selection of HEK293 Flp-In stable clones were carried out with hygromycin and the expression of the gene of interest verified through specific RT-PCRs.

Lentiviral particles encoding eGFP and U1 snRNA WT or ExSpe sm2 or sm17 or sm21 were prepared through cotransfection in HEK293T cells of psPAX vector encoding for gag-pol, tet, and rev proteins, pVSV-G encoding packaging proteins, and modified pLVTHM plasmid encoding the green fluorescent protein (GFP) under the cytomegalovirus (CMV) promoter and U1 snRNA WT or SMA-specific ExSpe U1 gene under their own promoter. Transfection efficiency was monitored through GFP evaluation at FACS. The viral supernatant was collected from transfected cells after 48 hr and concentrated by ultracentrifugation at 25,000 rpm for 90 min. The viral pellet was resuspended in PBS 1× and flash frozen in liquid nitrogen and aliquots were stored at −80°C until use. The titer of the virus we obtained was 4×10^8 bTU/ml. SMA fibroblasts were grown on 6-well plates and transduced with 4×10^6 lentiviral particles with a multiplicity of infection (MOI) of 15. AON 10–27 was transfected in HEK293 Flp-In cells as previously described.[32]

Administration of scAAV9 to *SMN2* Transgenic Mice

Mice were housed and handled according to institutional guidelines, and experimental procedures were approved by the International Centre for Genetic Engineering and Biotechnology (ICGEB) board, with full respect to the EU Directive 2010/63/EU for animal experimentation. A total of 12 *SMN2* transgenic mice (FVB.Cg-Tg(SMN2)89Ahmb *Smn1*^{tm1Msd}/J; Jackson Laboratories, stock number 005024), males or females, hemizygote or wild-type at the *Smn* locus were divided into three different groups and tested as follows: four mice were injected intraperitoneally (i.p.) at postnatal day 1 (P1) with 20 μl of scAAV9-ExSpe sm21 (titer = 2.23×10^{13} VG/ml, SignaGen Laboratories), four mice were injected with scAAV9-U1 WT (titer = 2×10^{13} VG/ml SignaGen Laboratories), and four untreated animals were used as controls. All the 12 mice were sacrificed after 4 weeks. Mouse tissues and organs (brain, heart, kidney, liver, skeletal muscle) were collected, snap frozen in liquid nitrogen, and stored at −80°C. For RNA extraction, 1 ml of Trizol (Invitrogen) was used to homogenize 100 mg of each tissue. Total RNA was then extracted according to the manufacturer's instructions.

RT-PCR, Sybr-Green qPCR, and TaqMan Assay

Total RNA was isolated with TRIzol reagent (Invitrogen). Reverse transcription was performed with random primers via SuperScript III reverse transcriptase (Invitrogen). Semiquantitative RT-PCR on SMA type I primary fibroblasts and endogenous HEK293 SMN transcripts was carried out with 5′-FAM-labeled E6-Fw and E8-467-Rev primers (Table S1 available online). RT-PCR on *SMN2* transgene in mice was carried out with a set of human-specific primers as previously reported.[31] RT-PCR after minigene splicing assay was performed as previously described.[12] For HEK293 cells and SMA fibroblasts, amplified products were digested with *DdeI* enzyme to discriminate transcripts coming from *SMN1* or *SMN2*. The resulting amplified products were run in denaturing capillary electrophoresis. The peaks resulting from capillary electrophoresis were analyzed through PeakScanner software and the ratio between splicing isoforms shown as histograms. The intensity of the bands in agarose gels was determined with ImageJ software. For specific detection of ExSpe U1 sm17 and sm21, total RNA was treated with DNase and RT-PCRs were carried out with U1sm17-Fw or U1sm21-Fw along with U1-160 Rev (Table S1). The efficiency (Eff) of the U1 WT, ExSpe U1 sm17, and sm21 qPCR reactions has been calculated by the following equation: Eff = 10(−1 / slope) − 1. TaqMan Assay on SMN E7 or ΔE7 was performed as previously described.[33] Sybr-green-based quantitative PCR on SMN total transcripts and SMN exon 2 or exon 7 splicing

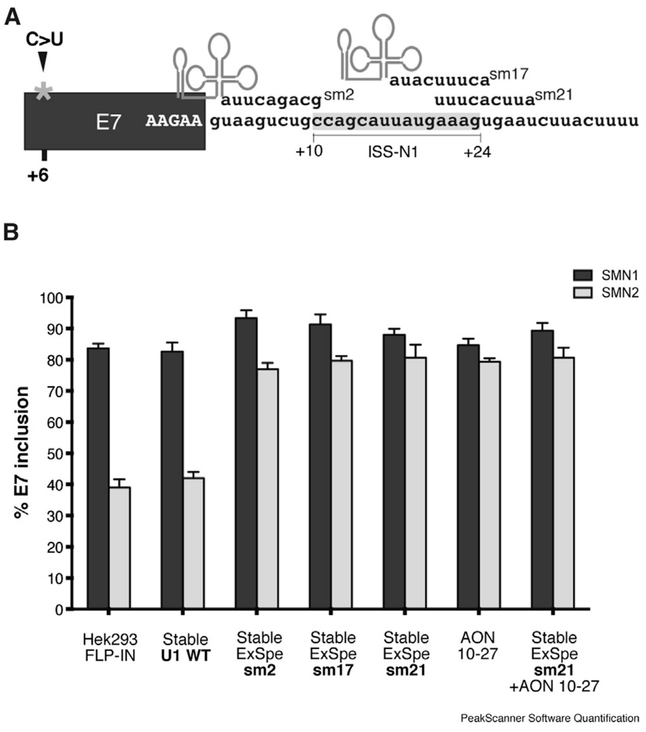

Figure 1. A Single Chromosome-Integrated Copy of *ExSpe U1* Improves *SMN2* E7 Inclusion in HEK293 Flp-In Stable Clones

(A) Schematic representation of *SMN* exon 7 pre-mRNA. The dark box represents the exon. The asterisk indicates the position of the exonic C-to-U transition in position +6. The sequence of the modified 5′ tails of the different ExSpe U1s and the target sequences on pre-mRNA are indicated. The hnRNPA1-dependent intronic splicing silencer N1 spanning from position +10 to +24 (ISS-N1) is shown.

(B) Total RNA extracted from the indicated cells was analyzed by RT-PCR with E6-F 5′-FAM-labeled and E8-467R primers. Amplification products were digested with *DdeI* restriction enzyme and the resulting fragments run on denaturing capillary electrophoresis for quantification of *SMN1* and *SMN2* isoforms. The ratio between the peaks corresponding to *SMN1* or *SMN2* spliced isoforms was calculated with Peak Scanner software. The percentage of exon 7 inclusion is expressed as mean ± SD of three independent experiments.

intermediates was carried out with Ex2a Fw in combination with Ex2b Rev, Int1_13850Fw in combination with Int2a_14006 Rev, and Int6 Fw in combination with Int7 Rev (Table S1).

mRNA Stability Assay

HEK293 Flp-In cells were treated with α-ammannitin (20 μg/ml) and collected every 5 hr at five different time points (0, 5, 10, 15, and 20 hr). Total RNA was extracted and treated with DNase, and Sybr-green-based quantitative RT-PCR on SMN total transcripts and SMN E7 splicing intermediates was carried out as reported above. 18S RNAs were used as normalization control with 18S Fw and 18S Rev primers (Table S1).

3′ Rapid Amplification of cDNA Ends Assay

Total RNA from HEK293 Flip-In stable clone lines expressing ExSpe U1 sm2, sm17, sm21, and the U1 snRNA WT was extracted with TRIzol reagent (Invitrogen). Reverse transcription was performed with oligo dT via SuperScript III reverse transcriptase (Invitrogen). 3′ RACE PCR was performed with forward primer located on SMN exon 6, SMN exon 7, or SMN exon 8, and Anchor primer as reverse (Table S1).

Immunoblot

Protein samples separated by NuPAGE 4%–12% Bis-Tris precast gels (Life Technologies) were electroblotted onto nitrocellulose membranes. The membrane was probed with monoclonal anti-SMN (BD Transduction Laboratories) and anti-α-tubulin (courtesy of Dr. Muro) as normalization control, followed by horseradish-peroxidase-conjugated rabbit anti-mouse secondary antibody. Protein signals were detected with SuperSignal West Femto Maximum Sensitive Substrate (Thermo Scientific).

Statistical Analysis

The data are expressed as the mean ± SD of different experiments done in triplicate. Data were compared with one-way ANOVA test. The p values are indicated in the figures and legends.

Results

A Single Copy of *ExSpe U1* Increases SMN Exon 7 Inclusion in HEK293 Flp-In Cells

We previously reported three ExSpe U1s, named sm2, sm17, and sm21, active on *SMN2* exon 7 (E7) minigene.[12] They bind to 9-bp-long intronic sequences located 2, 17, and 21 bases downstream of E7 donor site and partially overlap with the previously described hnRNPA1-dependent intronic splicing silencer N1 (ISS-N1),[31] which is the target of the antisense oligonucleotide (AON) 10–27 (Figure 1A). To understand the effect of ExSpe U1s on SMN pre-mRNA processing, we took advantage of the HEK293 Flp-In system. In these cells, the insertion of a unique copy of the *ExSpe U1s* at a specific FRT chromosomal site allowed us to evaluate their effect on endogenous SMN E7 pre-mRNA processing and to perform a reliable comparison between the different ExSpe U1s. We created HEK293 Flp-In stable clones expressing *ExSpe U1s* sm2, sm17, or sm21, as well as *U1 snRNA WT* as control. We analyzed at least three different clones for each group and we did not observe any significant effect on cell viability and proliferation (Figure S1), suggesting that in these experimental conditions, ExSpe U1s have no toxic effect on cultured cells. Total RNAs from stable clones were analyzed by RT-PCR and the resulting amplified fragments digested with *DdeI* restriction enzyme to discriminate between the splicing products derived from endogenous *SMN1* and *SMN2*. Semiquantitative RT-PCR analysis in stable clones expressing ExSpe U1s showed a significant increase in the percentage of *SMN2* exon 7 inclusion from ~45% up to ~80%–85% (Figures 1B and S2). The same level of *SMN2* E7 inclusion was obtained with the AON 10–27 treatment (Figures 1B and S2). As expected, ExSpe U1 and AON also affected *SMN1*, inducing an increase in the *SMN1* E7 inclusion (Figure 1B). Although HEK293 cells produce high levels of SMN protein because

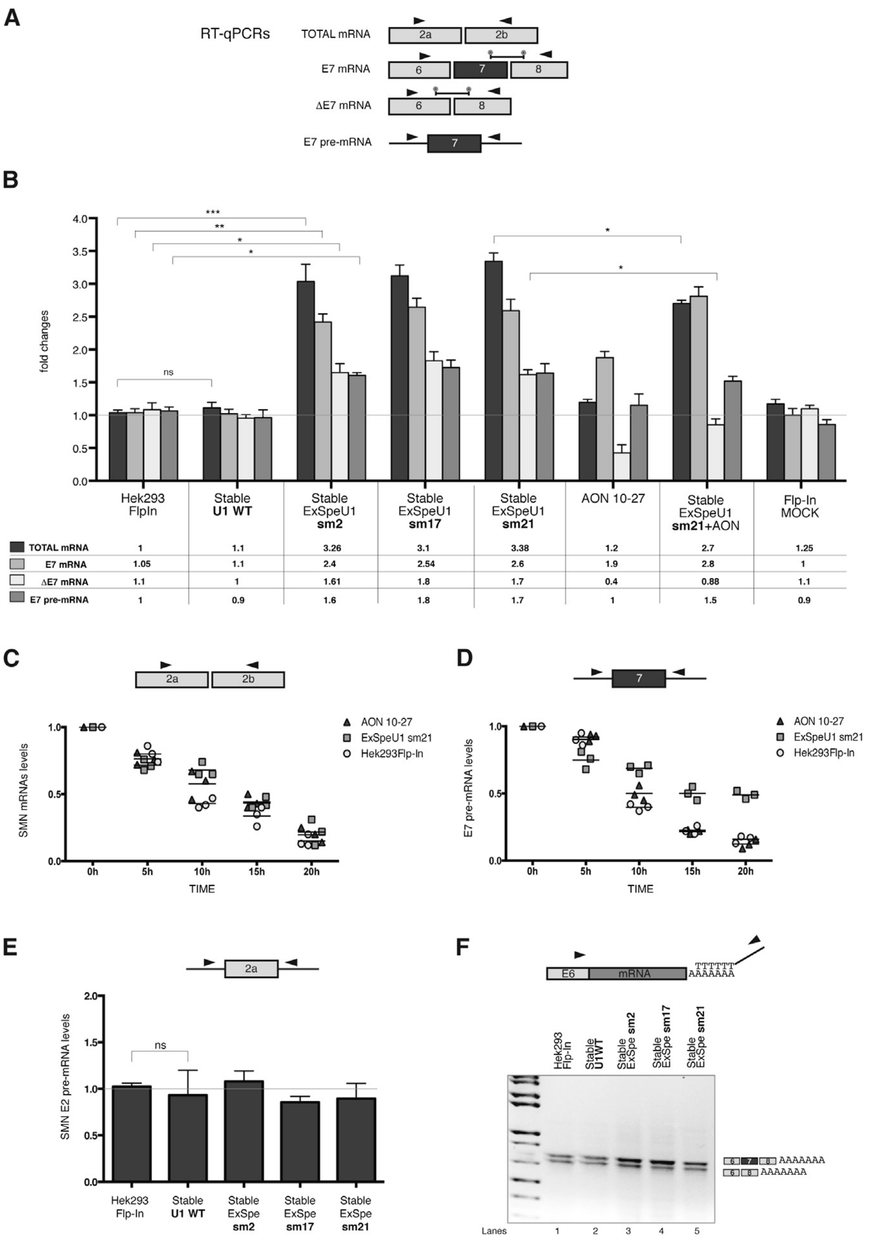

	Hek293 FlpIn	Stable U1 WT	Stable ExSpeU1 sm2	Stable ExSpeU1 sm17	Stable ExSpeU1 sm21	AON 10-27	Stable ExSpeU1 sm21+AON	Flp-In MOCK
TOTAL mRNA	1	1.1	3.26	3.1	3.38	1.2	2.7	1.25
E7 mRNA	1.05	1.1	2.4	2.54	2.6	1.9	2.8	1
ΔE7 mRNA	1.1	1	1.61	1.8	1.7	0.4	0.88	1.1
E7 pre-mRNA	1	0.9	1.6	1.8	1.7	1	1.5	0.9

Figure 2. ExSpe U1s Increase SMN Transcripts in HEK293 Flp-In Stable Clones and Stabilize a Pre-mRNA Splicing Intermediate

(A) Schematic representation of the SMN mRNAs and pre-mRNA analyzed through quantitative RT-PCR. Boxes are exons and lines are introns. The localization of the primers is indicated with arrows. The specific probes used for E7 and ΔE7 TaqMan assay are indicated with lines.

(B) Total RNA from the indicated cells was extracted. Total SMN mRNA and SMN E7 splicing intermediate were evaluated through quantitative sybr-green-based RT-PCRs, and E7 and ΔE7 mRNAs through a specific TaqMan assay. Each RNA molecule level was set to 1 in HEK293 Flp-In cells and the variation of the analyzed mRNAs and pre-mRNA expressed as fold changes. The results are expressed as mean ± SD of three different experiments performed in triplicate. ***$p < 0.001$, **$p < 0.01$; ns, not significant. One-way ANOVA test.

(C and D) SMN mRNA and pre-mRNA stability assay. HEK293 Flp-In cells treated or not with AON 10–27 and HEK293 Flp-In sm21 stable clone were treated with 20 μg/ml (0h). Cells were harvested at four different time points after 5 hr (5h), 10 hr (10h), 15 hr (15h), and 20 hr (20h). Total RNA was extracted and quantitative RT-PCR carried out with the primers indicated at the top of each scatter plot. 18S levels were used as normalization control.

(E) Total RNA was extracted from the indicated cells and exon 2 splicing intermediate levels evaluated through qRT-PCR as indicated in the top of the panel. The results are expressed as mean ± SD of three different experiments performed in triplicate. ***$p < 0.001$, **$p < 0.01$; ns, not significant. One-way ANOVA test.

(F) 3′-RACE (rapid amplification of cDNA ends) assay. Total RNA was extracted from the indicated cells and retrotranscribed with an oligodT with an anchor. Polyadenylated transcripts from *SMN* were evaluated with Exon-6_Fw primer in combination with Anchor_Rev primer. The amplified fragments were separated on 1.5% agarose gel. The identity of each band, schematically reported on the right of the gel, has been verified through direct sequencing of PCR products.

of the presence of both *SMN1* and *SMN2*, we tried to detect changes in the SMN protein expression in HEK293 clones stably expressing ExSpe U1s. Interestingly, immunoblot analysis revealed a small but reproducible effect (~1.3- to 1.4-fold increase) in the level of SMN protein in HEK293 Flp-In cells expressing ExSpe U1s (Figure S3). The control stable clone expressing U1 snRNA WT affected neither SMN splicing (Figure 1B) nor SMN protein (Figure S3).

ExSpe U1s Have Multiple Effects of SMN Pre-mRNA Processing

Beyond its well-established role in splicing, the U1 snRNP has been shown to have additional effects on pre-mRNA processing.[7,9,34] Therefore, we consider the possibility that ExSpe U1 might result in a more complex effect on SMN processing.

To better unravel the effect of ExSpe U1 on SMN pre-mRNA processing, we evaluated several SMN transcripts in a quantitative manner with specific primers. We analyzed the mature SMN transcripts (total mRNA), transcripts with or without E7 (E7 and ΔE7 mRNAs, respectively), and the pre-mRNA. This analysis was performed in ExSpe U1 HEK293 clones and in cells treated with AON 10–27 (Figure 2A), all showing comparable changes in the percentage of exon 7 (E7) inclusion (Figure 1B). Analysis of the expression levels of ExSpe U1s sm17 and sm21 from the corresponding HEK293 Flp-In stable clones

showed that both ExSpe U1 sm17 and sm21 are expressed at the same level, that is ~100 time less in comparison to the endogenous U1 snRNA level (Figure S4). Interestingly, in all the stable clones expressing ExSpe U1s, but not in AON-treated cells, we observed up to a ~3.2-fold increase in total SMN mRNA (Figure 2A). A more detailed analysis of the mature transcripts with or without E7 showed that ExSpe U1s improved both SMN isoforms, with a more pronounced effect on the E7 isoform (Figure 2B). The E7 mRNA was increased to ~2.5-fold whereas the ΔE7 increased to ~1.7-fold (Figure 2B). Interestingly, targeting the ISS with the AON 10–27 did not increase the total amount of mRNA: in this case, the increased level of E7 isoform is associated with a corresponding reduction of the ΔE7 isoform (Figure 2B). To better clarify this aspect, we evaluated the abundance of pre-mRNA transcripts via primers located in introns across exon 7 and exon 2 (E2). The SMN E7 pre-mRNA—but not SMN E2 pre-mRNA (Figure 2E)—is increased ~1.7-fold in ExSpe U1 stable clones, whereas these pre-mRNAs are not affected in AON-treated cells. These results suggest that whereas the AON modulates splicing decision, ExSpe U1 exerts an additional effect on SMN pre-mRNA processing, which results in a general increase in both the SMN mRNAs and E7 pre-mRNA.

To investigate a possible synergic effect of AON and ExSpe U1, we compared the different SMN transcripts abundance in the ExSpe U1 sm21 stable clone with and without the AON 10–27 and in HEK293 Flp-In cells treated with the antisense oligo. In comparison to the AON treatment alone, AON and ExSpe U1 together resulted in a significant increase in all SMN transcripts (Figure 2B, compare AON 10–27 with stable ExSpe sm21+AON). On the other hand, the AON 10–27+ExSpe U1 treatment showed only a slight change in SMN mRNA and pre-mRNA in comparison to ExSpe U1 alone. In addition, the increase of the amount of E7 mRNA at the expense of the ΔE7 isoform is more pronounced in ExSpe U1 sm21 stable cells treated with AON 10–27 in comparison to ExSpe U1 sm21 untreated cells (Figure 2B, compare stable ExSpe sm21 lanes with stable ExSpe sm21+AON lanes). These findings suggest that ExSpe U1 and AON 10–27 cooperate synergically in the SMN splicing rescue.

Because the increased amount of total SMN mRNAs and E7 pre-mRNAs in ExSpe U1 stable clones could be due to increased transcript stability, we decided to block transcription with α-amanitin, a compound that specifically interferes with RNA polymerase II (RNA PolII). The analysis was performed on ExSpe U1 sm21 stable clone and in cells treated with AON 10–27 (Figures 2C and 2D). Quantitative RT-PCR showed no differences in the mature SMN transcripts decay rate between untreated cells and cells treated either with ExSpe U1 or with the AON (Figure 2B). In contrast, analysis of the SMN E7 pre-mRNAs showed that ExSpe U1 stable clones had a ~25%–30% lower decay rate in comparison to cells treated with the AON or to normal cells (Figure 2D). In fact, 20 hr after inhibition of transcription, the ExSpe U1 cells retain ~40% of SMN E7 pre-mRNAs whereas the AON-treated or normal cells have ~10% (Figure 2D). Thus, ExSpe U1s promote E7 inclusion and inhibit the decay of the SMN E7 pre-mRNA.

To investigate the possibility that ExSpe U1s might protect the SMN transcripts from a premature polyadenylation, we performed a 3′-RACE. By using a forward primer located on SMN exon 6, we detected two transcripts—with and without exon 7—that use a unique polyadenylation signal (PAS) within exon 8 (Figure 2F). As expected, ExSpe U1 increases the ratio between E7/ΔE7 isoforms but did not affect poly(A) sites. 3′-RACE assay with forward primers on exon 7 or 8 confirmed the usage of the canonical poly(A) site (Figure S5).

ExSpe U1s Replace Endogenous U1 snRNP in SMN Exon 7 Definition

To further investigate how ExSpe U1 rescues E7 skipping and facilitates SMN processing, we considered the roles of the inhibitory splicing factor hnRNPA1 that binds to the ISS-N1 and of the endogenous U1 snRNP. Both overexpression of hnRNPA1 and functional suppression of U1 snRNP inhibit *SMN2* E7 inclusion.[35] We tested in *SMN2* E7 minigene the dose-dependent effect of hnRNPA1 overexpression on the rescue efficiency mediated by ExSpe U1s or by AON 10–27. Accordingly to the proposed antisense mechanism, cotransfection of hnRNPA1 did not affect the splicing rescue induced by the oligonucleotide, indicating that it directly and efficiently blocks the binding of the splicing factor to the ISS (Figure 3C, lanes 5–8). On the contrary, the increase in the amount of the cotransfected splicing factor progressively reduced the splicing rescue mediated by ExSpe U1 sm21 (Figure 3C, lanes 9–12). Similar results were obtained with the other two ExSpe U1s, sm2 and sm17 (data not shown). We also explored the role of endogenous U1 snRNP performing *SMN2* minigene splicing assays by using a U1 snRNA-specific decoy, D1. The D1 plasmid encodes for a decoy RNA complementary to the 5′ tail of the endogenous U1 snRNA (Figure 3A, left) and was previously reported to induce skipping of *SMN2* E7.[35] We cotransfected decoy plasmids along with ExSpe U1s or AON 10–27 and evaluated the resulting effect on *SMN2* splicing pattern (Figure 3B). The U1 snRNA decoy (D1) differently affected the rescue efficiency of AON 10–27 and ExSpe U1 sm21. Specifically, D1 reduced the percentage of *SMN2* E7 inclusion induced by the oligonucleotide from 100% inclusion to 25% (Figure 3B, lanes 5 and 6), whereas it has no effect on ExSpe U1 sm21 (lanes 4 and 8). The other two ExSpe U1s, sm2 and sm17, were also not affected by D1 treatment (data not shown). The control D3 plasmid (a decoy RNA that contains a mismatch in the base pairing with the endogenous U1; Figure 3A, right) did not affect the splicing patterns. Thus, in contrast to AON 10–27, ExSpe U1s are sensitive

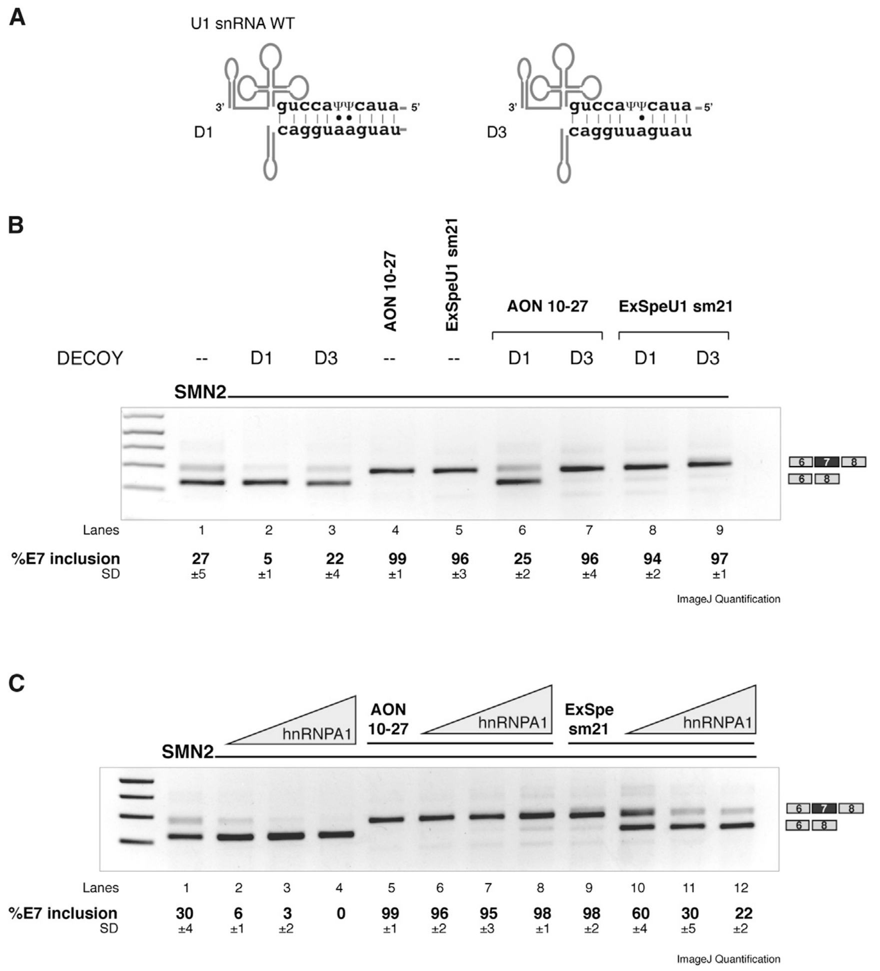

Figure 3. ExSpe U1s Do Not Require the U1 snRNP and Are Sensitive to hnRNP A1 Overexpression

(A) Schematic representations of the base pairing between the 5′ tail of the U1 snRNA WT and the tail of the RNA U1 decoy (D1) or with the tail of the RNA U1 mismatch decoy (D3).

(B) HEK293 cells were transfected with pCI SMN2 plasmid alone or in combination with plasmids encoding for RNA U1 decoy (D1), RNA U1 mismatch decoy with a variation in position +3 (D3), ExSpe U1 sm21, or AON 10–27. Total RNA was extracted and retrotranscribed with random primers. RT-PCR was performed with pCIF Fw and E8-75 Rev primers. The resulting amplified products were separated on 2% agarose gel. The identity of the bands is shown. Band intensity was quantified with ImageJ software and the percentage of exon inclusion is reported for each lane at the bottom of the gel, as mean ± SD of two different experiments.

(C) SMN2 minigene splicing assay has been performed in HEK293 cells cotransfected with ExSpe U1 sm21 (lanes 9–12) or AON 10–27 (lanes 5–8) along with increasing amount of hnRNPA1 splicing factor (50-150-250 ng, lanes 10–12 and 6–8, respectively). Total RNA was extracted and RT-PCR carried out as described in (B). The identity of the bands is indicated on the right of the panel and their intensity has been quantified with ImageJ software. The percentage of exon 7 inclusion, expressed as mean ± SD of three different experiments, is reported below the gel.

to hnRNPA1 overexpression but do not require the presence of the endogenous U1 snRNP at the E7 donor splice site. Therefore, ExSpe U1 mainly replaces the function of endogenous U1 snRNP in exon definition and does not act with antisense mechanism on the hnRNPA1-dependent ISS.

Lentiviral Transduction of ExSpe U1s in SMA Type I Fibroblasts Rescues SMN2 Exon 7 Splicing Pattern and Increases SMN Protein Levels

To determine the possible therapeutic activity of ExSpe U1s, we used SMA type I primary fibroblasts that lack *SMN1* and have only two copies of *SMN2*. Fibroblasts were infected with lentiviral particles expressing the three different noncoding ExSpe U1 RNAs or the normal U1 snRNA WT as control, along with the green fluorescent protein (GFP), that was used for the evaluation of cell transduction efficiency. Lentiviral infection resulted in the expression of the corresponding ExSpe U1 RNAs (Figure S6). After lentiviral infection, we evaluated the percentage of *SMN1* and *SMN2* E7 inclusion and the total amount of E7 and ΔE7 SMN isoforms. In SMA fibroblasts, lentiviral transduction with the ExSpe U1s sm2, sm17, and sm21 corrected the splicing defect. The percentage of *SMN2* E7 inclusion increased from the basal level of ~38% up to ~80%, which corresponds to the *SMN1* value observed in control fibroblasts (Figure 4A). Infection with U1 snRNA WT did not affect the splicing pattern. Evaluation of the E7 and ΔE7 SMN isoforms showed a ~2.5-fold increase of the E7-corrected isoform in comparison to untreated cells (Figure 4B). Consistent with a complex effect on pre-mRNA processing, ExSpe U1s increased also the SMN ΔE7 isoform, although to a lesser extent. To assess whether *SMN2* splicing rescue in SMA fibroblasts resulted in changes at the protein level also, we detected the SMN protein through immunoblot (Figures 4C and 4D). Quantification of SMN band intensity showed that the three ExSpe U1s promoted respectively a ~1.65-, 1.8-, and 1.9-fold increase in the SMN protein in comparison to untreated SMA fibroblasts. Interestingly, the protein rescue obtained with ExSpe U1s reaches the SMN level present in control fibroblasts (Figure 4D).

AAV-Mediated Delivery of ExSpe U1 sm21 Enhances *SMN2* Splicing in Transgenic Mice

To evaluate the ExSpe U1 activity in vivo, we decided to test its effect on *SMN2* splicing in mice harboring the

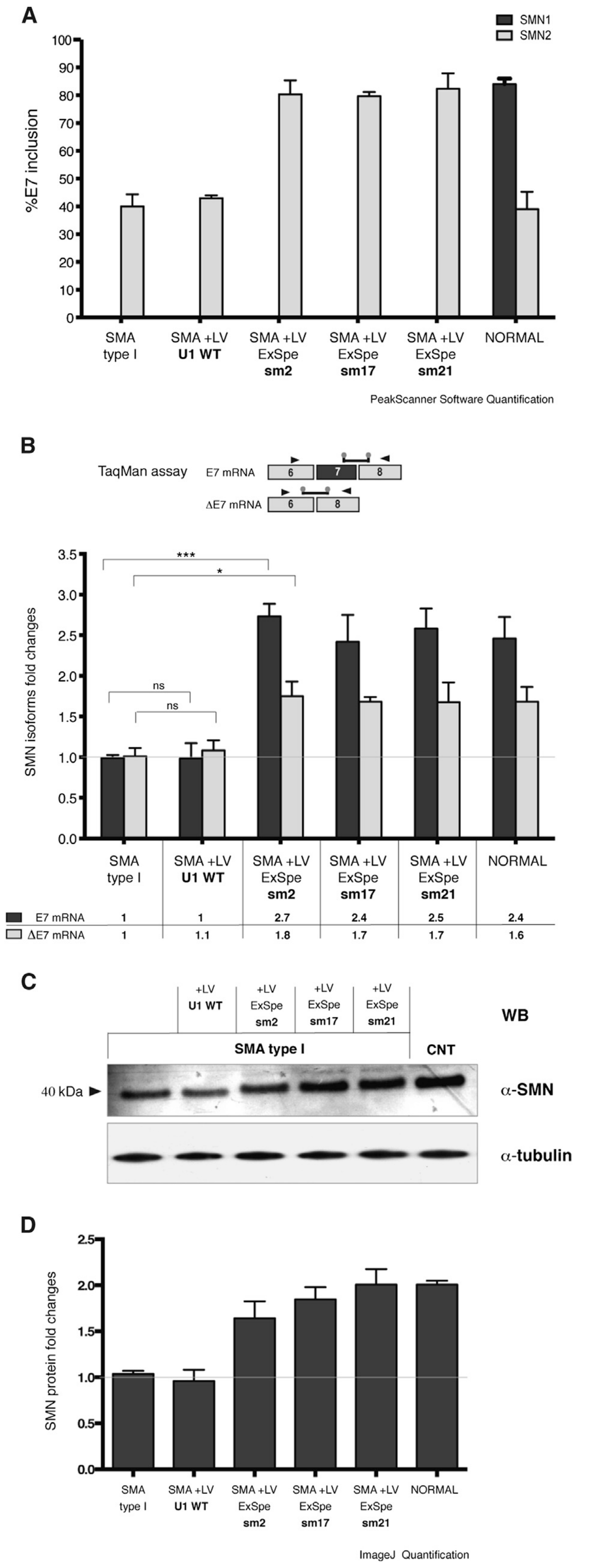

Figure 4. Lentiviral-Mediated Transduction of ExSpe U1s Rescues SMN2 Exon 7 Splicing and Increases Cellular Levels of SMN Protein

(A) SMA type I fibroblasts (G3813, Coriell Institute) were transduced with lentiviral particles expressing ExSpe U1s sm2, sm17, or sm21, as well as U1 WT, with a MOI of 15. Normal fibroblasts (G3814, Coriell Institute) were used as control. Transduction efficiency was evaluated through GFP-positive cells at confocal microscope. Total RNA was extracted and RT-PCR carried out with a 5′-FAM-labeled E6 Fw along with E8-467 Rev primers. The resulting amplified fragments were digested with *DdeI* restriction enzyme to distinguish *SMN1* and *SMN2* products and run in capillary electrophoresis. The peaks were analyzed with PeakScanner software and the data are reported as histograms that express the percentage of exon 7 inclusion as mean ± SD of three independent experiments.

(B) The RNA samples described in (A) were analyzed also by quantitative TaqMan Assay. Two probes for full-length (E7) or delta7 (ΔE7) SMN isoforms were used along with FL-Fw/FL-Rev and ΔE7-Fw and ΔE7-Rev set of primers, as depicted in the upper part of the panel. 18S RNA was used as normalization control. The levels of E7 and ΔE7 in SMA type I fibroblasts were set to 1. The histograms express the fold changes in SMN E7 or ΔE7 isoforms in SMA fibroblasts expressing ExSpe U1s or U1 WT compared to untreated ones and to normal fibroblasts. ***$p < 0.001$, **$p < 0.01$; ns, not significant. One-way ANOVA.

(C) SMA type I fibroblasts (Coriell Institute, 3813) were transduced with lentiviral particles as described in (B). At 72 hr after transduction, cells were harvested and protein samples loaded on 4%–12% polyacrylamide gel. The SMN protein was detected by immunoblotting in SMA type I fibroblasts (lane 1) and normal control fibroblasts (lane 6). SMN protein in SMA fibroblasts treated with U1 WT or ExSpe U1s sm2, sm17, or sm21 are shown in lanes 2 and 3–5, respectively. The same blot was reprobed with an antibody against tubulin as loading control.

(D) The intensity of SMN bands reported in (C) was quantified with ImageJ software. The histograms indicate the SMN protein fold increase in comparison to the value of SMA-untreated fibroblasts (set to 1), expressed as mean ± SD of three independent experiments.

SMN2 transgene. We used self-complementary adeno-associated viruses serotype 9 (scAAV9) encoding for the U1 snRNA WT (scAAV9-U1WT) or the ExSpe U1 sm21 (scAAV9-ExSpe U1 sm21). Recipient mice of both sexes were transgenic for *SMN2* and hemizygotes or WT at the mouse *Smn* locus. We injected four different *SMN2* transgenic mice intraperitoneally at postnatal day 1 (P1) with scAAV9-ExSpe U1 sm21. As controls, four animals were treated with scAAV9-U1WT. The mice were sacrificed 30 days after AAV administration and total RNA was extracted from brain, heart, kidney, liver, and skeletal muscle. We analyzed *SMN2* E7 splicing pattern and corresponding E7 and ΔE7 isoforms via SMN human-specific primers (Figure 5). In all tissues analyzed, we observed in mice treated with ExSpe U1 sm21 a significant increase of the percentage of *SMN2* E7 inclusion and in the amount of the corresponding E7 isoform. In these animals, the percentage of E7 inclusion increased from ~10% up to ~45%–50% (Figures 5A–5E, left, lanes 1–4) and the total amount of E7 isoform showed up to ~2.5- to 3-fold increase. Interestingly, as observed in stable Flip-In clones and SMA fibroblasts, the increased levels of the E7 mRNAs do not associate with decreases in the ΔE7 mRNA. In fact, the levels of ΔE7 remained stable or even slightly increased in most tissues analyzed (Figure 5, right). The animals injected with the AAV9-U1WT did not

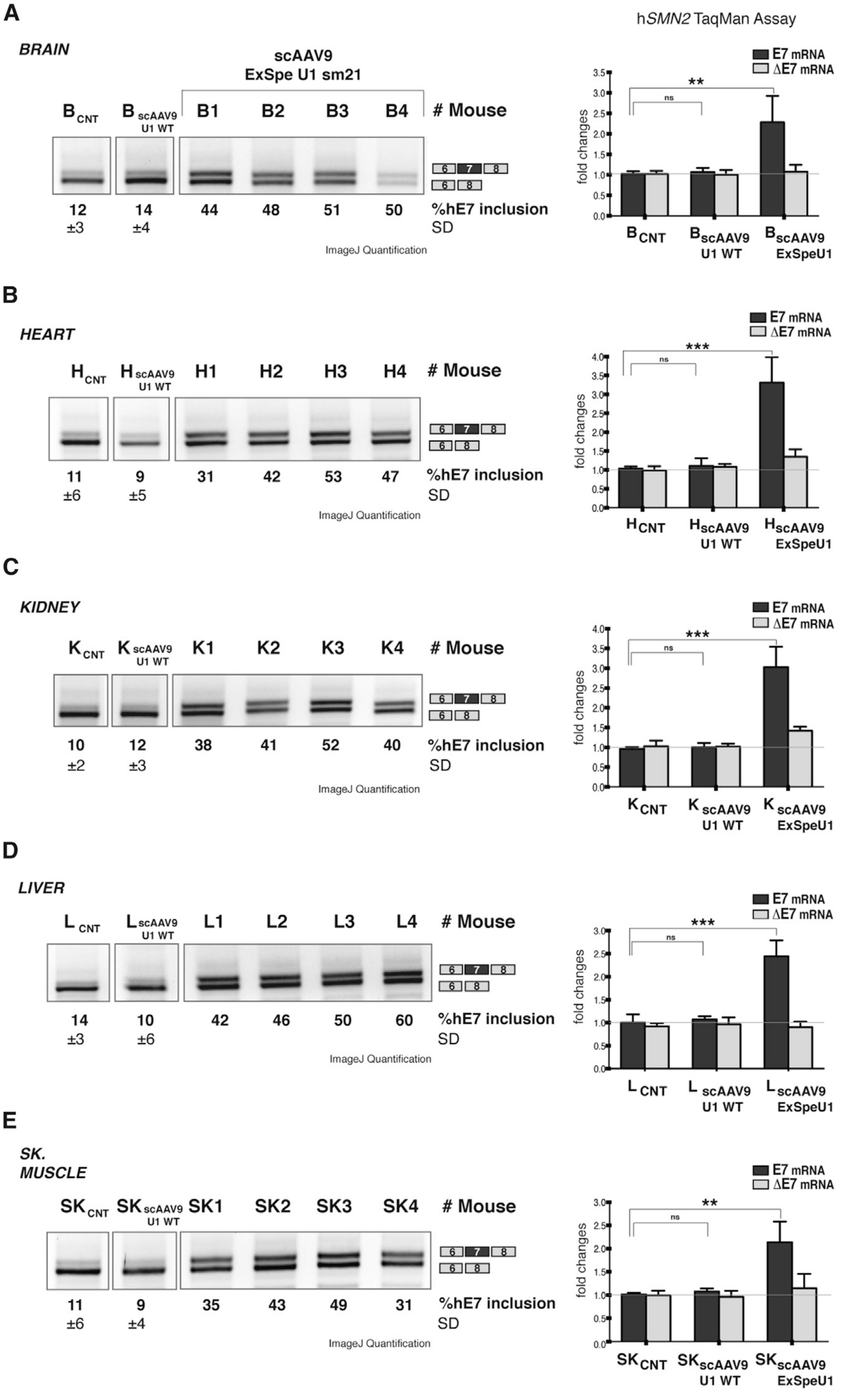

Figure 5. Effect of ExSpe U1 sm21 in *SMN2* Transgenic Mice

Four different *SMN2* transgenic mice (Jackson Laboratories, stock 005024) of both sexes were injected intraperitonelly at P1 with scAAV9-Exspe U1 sm21 (animals 1–4), four were injected with scAAV9-U1 WT, and four animals were not treated and have been used as controls. Mice were sacrificed 4 weeks after AAV administration. Organs and tissues including brain (A), heart (B), kidney (C), liver (D), and skeletal muscle (E) were harvested and total RNA was extracted with Trizol reagent. Left: semiquantitative RT-PCR was carried out with a set of *SMN2*-specific primers. The resulting amplified products were solved on 1.5% agarose gels. Lanes 1 and 2 show the pattern of splicing of uninjected (CNT) and scAAV9-U1WT-injected mice, as well as the percentage of human exon 7 (hE7) inclusion expressed as mean ± SD of the four different mice belonging to each group. Lanes 3–6 show the single pattern of splicing of hE7 of the four animals (mouse 1–4) injected with scAAV9 ExSpe U1 sm21. The identity of the bands is depicted on the right of the gels and the percentage of exon inclusion for each lane, quantified by ImageJ software, is reported. Right: human-specific SMN TaqMan assay to detect the levels of E7 or ΔE7 isoforms. The levels of E7 or ΔE7 in the untreated *SMN2* transgenic mouse (group 1, control) were set to 1. The histograms express the fold changes of hSMN2 E7 or ΔE7 isoforms in group 2 and group 3 animals (treated with U1 WT or ExSpe U1 sm21, respectively) compared to the untreated control group. Error bars show standard deviations (***$p < 0.001$, **$p < 0.01$, *$p < 0.1$; ns, not significant. One-way ANOVA test).

show any change. These data demonstrate that ExSpe U1 sm21 is able to correct aberrant *SMN2* mRNAs in vivo, suggesting the reliability of this approach for a possible therapeutic application for SMA.

Discussion

We have previously proposed a strategy based on ExSpe U1s to correct exon-skipping defects. These molecules are derivatives of U1 snRNA and bind by complementarity of their modified 5′ tail to intronic sequences located at different distances downstream of the 5′ ss, the canonical U1 snRNP binding position. ExSpe U1s promote the inclusion of different defective exons in minigene systems.[12] To establish their therapeutic potential and to elucidate their mechanism of action, we have investigated the effect of ExSpe U1s by using SMA cellular models and mice harboring the *SMN2* transgene. The results we obtained demonstrate that ExSpe U1s are operative in SMA type I fibroblasts where they promote E7 inclusion and increase SMN protein levels. They are also effective in vivo, correcting *SMN2* aberrant splicing in mice. Low ExSpe U1 amounts are needed and no toxicity is apparent. ExSpe U1s show a positive effect on SMN pre-mRNA processing, which is different from and complements that of AONs.

ExSpe U1s Have a Complex Effect of SMN Pre-mRNA Processing

To investigate the effect of ExSpe U1s on *SMN* pre-mRNA processing, we analyzed the percentage of SMN E7 inclusion as well as the amount of E7, ΔE7, total mRNAs, and E2 and E7 pre-mRNAs derived from *SMN*. Our data indicate that ExSpe U1s have two main effects on *SMN* processing, namely to increase the amount of E7 pre-mRNA substrate and to promote the correct selection of the defective exon. In comparison with normal cells, the stable clones expressing ExSpe U1s from a single chromosome-integrated gene have 1.5-fold more *SMN* E7 pre-mRNA intermediate and three times more total *SMN* mRNA (Figure 2B). Interestingly, we did not detect any difference in *SMN* E2 pre-mRNA intermediate levels between HEK293 Flp-In and stable clones expressing different ExSpe U1s (Figure 2E), strongly suggesting that the increase in the *SMN* E7 pre-mRNA and mRNA does not reflect an enhancement of the transcription. In addition, the increase of E7 pre-mRNA levels does not appear to be related to changes in splicing kinetics, as in this latter case the total amount of *SMN* mRNA should not be affected. Moreover, this effect is specific for ExSpe U1s, as shown by the fact that the antisense oligonucleotide that targets the corresponding intronic sequences did not increase either the total *SMN* mRNA or pre-mRNA transcripts. Analysis of the E7 and ΔE7 mRNAs showed that the AON enhances E7 levels at the expense of ΔE7, consistent with a "pure" effect on the splicing decision, as previously reported.[31] In contrast, the higher levels of the pre-mRNA substrate induced by ExSpe U1s comprise both isoforms, with a more pronounced effect on the E7 mRNAs (Figure 2B). The increase in E7 and ΔE7 mRNAs is evident in the HEK293 Flp-In cells, in lentiviral-infected SMA type I fibroblasts (Figure 4B), and partially in vivo on the *SMN2* transgene in AAV-treated mice (Figures 5A–5E, right). Thus, our results demonstrate that the ExSpe U1 RNA molecule corrects splicing and also increases mRNA and pre-mRNA intermediates. The effect on pre-mRNA processing is probably due to the fact that, whereas the AON acts as a masking molecule, ExSpe U1s recruit splicing factors on the defective upstream exon and diminishes the effect of the mutation. In fact, we observed that overexpression of hnRNPA1 does not affect the *SMN2* splicing rescue mediated by the oligonucleotide (Figure 4). On the contrary, the splicing rescue promoted by ExSpe U1 was reduced by hnRNPA1 overexpression. On the other hand, functional inactivation of U1 snRNP had no effect on ExSpe U1 but abolished the AON-dependent rescue (Figure 3B). Thus, AON and ExSpe U1s act through different mechanisms: AON as an antisense molecule masking the ISS-N1, and ExSpe U1s promoting per se exon definition, most likely by recruiting factors important for splicing. The effect of ExSpe U1s on SMN transcript abundance and on the stability of the corresponding pre-mRNA indicates that, in addition to their effect on splicing, ExSpe U1s affect other pre-mRNA processing steps. Actually, U1 snRNP has been shown to protect pre-mRNA premature termination at cryptic polyadenylation sites within introns[7–9] and to regulate transcriptional efficiency in model systems.[36,37] However, our 3′-RACE analysis did not reveal any change in the length of 3′ UTR or usage of alternative polyadenylation sites in ExSpe U1-expressing cells (Figures 2F and S5), indicating that ExSpe U1s do not affect SMN polyadenylation.

ExSpe U1s as Therapeutic Tools for Splicing Modulation in SMA

In addition to the positive effect on pre-mRNA processing, ExSpe U1s exhibit additional properties regarding an efficient splicing correction. A crucial factor in gene transfer strategies is the copy number of the therapeutic gene that every single cell has to reach. For example, at least 100 copies per nucleus of the U7-antisense encoding gene are required to promote a therapeutic exon skipping in vivo in Duchenne muscular dystrophy (DMD [MIM 310200]).[38] Taking advantage of the well-controlled HEK293 Flp-In cell system, in the present work we demonstrate that a single copy of *ExSpe U1* sm17 or sm21 gene leads to the expression of comparable levels of the corresponding molecule among different stable clones. Interestingly, the very low relative amount of ExSpe U1 versus endogenous U1 snRNA (<1%) present in stable clones (Figure S4) corrects with the same efficacy *SMN2* E7 splicing and increases total SMN mRNAs (Figure 2B). The levels of exon inclusion obtained with a single integrated copy are comparable to the effect obtained after lentiviral infection of SMA fibroblasts and are in line with other therapeutic strategies.[39–42] The significant rescue activity observed with a single copy of ExSpe U1 might be due to the strong constitutive ubiquitous U1 promoter or to a facilitated cotranscriptional recruitment of the resulting particle on the CTD of RNA polymerase II, as reported for normal U1 snRNP.[37,43]

It is important to note that the ExSpe U1-mediated increase of the ΔE7 isoform we observed in SMA fibroblasts, in stable HEK293 Flp-In clones and, to a lesser extent, also in *SMN2* transgenic mice is likely to result in an additional therapeutic advantage because even a modest *SMN* ΔE7 increase has been shown to be beneficial in SMA.[44] In fact, although the ΔE7 isoform contains a strong degradation signal and is therefore highly unstable,[28] it retains some functional activity in cells.[45] Furthermore, expression of multiple copies of SMN ΔE7 cDNAs transgene in mice clearly reduces SMA severity.[44]

An important feature of ExSpe U1s is that, contrary to modified U1 snRNA that directly binds to the 5′ ss in *SMN2*[46] or in *coagulation FVII* exon 5,[47] they are shown not to be toxic, at least to cells in culture (Figure S1). Apart from the invariant *gt* dinucleotide, the other nucleotides of mammalian donor splice site sequence differ from the consensus sequence, particularly in alternatively spliced exons. It is therefore possible that modified U1s binding to the 5′ ss elicit a toxic effects, affecting the fine regulation of some critical exons. ExSpe U1s reduce this possibility by

targeting nonconserved intronic sequences distant from the 5′ ss. However, future studies will be required to evaluate potential off-target effects in vivo in animal models.

As an in vivo proof of ExSpe U1 molecule properties, we have showed that AAV-mediated administration of ExSpe U1 in *SMN2* transgenic mice recovers E7 inclusion from ~15% up to 50% in brain, heart, kidney, liver, and skeletal muscle (Figure 2). This result, along with the previous analysis of FVII deficiency (MIM 227500),[47] demonstrates that ExSpe U1 can be efficiently expressed in vivo in different mouse tissues (Figure 5) and exert an effect on its natural human *SMN2* target. The *Smn*-null transgenic mice we have used have a severe phenotype and survive only a few days.[48] Studies are in progress to establish either the rescue at the protein level or the effect on survival and phenotypic rescue in the less severely affected *SMN2* transgenic mice.

In conclusion, we provide in vitro and in vivo complementary evidence indicating a strong therapeutic activity of ExSpe U1s that correct exon skipping in the SMA model. Because this type of splicing error represents a significant percentage of disease-causing mutations in humans, ExSpe U1s will have to be explored in relationship with different pathologies as potential tailored tools aimed at correcting pre-mRNA splicing defects.

Supplemental Data

Supplemental Data include six figures and one table and can be found with this article online at http://dx.doi.org/10.1016/j.ajhg.2014.12.009.

Acknowledgments

We thank Rodolfo Garcia and Marco Baralle for the proofreading of the manuscript, Maurizio Romano for the help with the statistic analysis, Alessandro Marcello for the helpful discussions on lentiviral vectors, and Adrian Krainer and Xavier Roca for U1 snRNA decoy and pCI-SMN2 plasmids. This work was supported by the Association Francais contre les Myophaties (AFM) (grant number 16101) and Telethon foundation (grant number GGP14190).

Received: September 24, 2014
Accepted: December 5, 2014
Published: December 31, 2014

Web Resources

The URL for data presented herein is as follows:

Online Mendelian Inheritance in Man (OMIM), http://www.omim.org/

References

1. Pagani, F., and Baralle, F.E. (2004). Genomic variants in exons and introns: identifying the splicing spoilers. Nat. Rev. Genet. *5*, 389–396.
2. Pagani, F., Stuani, C., Tzetis, M., Kanavakis, E., Efthymiadou, A., Doudounakis, S., Casals, T., and Baralle, F.E. (2003). New type of disease causing mutations: the example of the composite exonic regulatory elements of splicing in CFTR exon 12. Hum. Mol. Genet. *12*, 1111–1120.
3. Wahl, M.C., Will, C.L., and Lührmann, R. (2009). The spliceosome: design principles of a dynamic RNP machine. Cell *136*, 701–718.
4. Roca, X., Krainer, A.R., and Eperon, I.C. (2013). Pick one, but be quick: 5′ splice sites and the problems of too many choices. Genes Dev. *27*, 129–144.
5. Egloff, S., O'Reilly, D., and Murphy, S. (2008). Expression of human snRNA genes from beginning to end. Biochem. Soc. Trans. *36*, 590–594.
6. Merkhofer, E.C., and Johnson, T.L. (2012). U1 snRNA rewrites the "script". Cell *150*, 9–11.
7. Kaida, D., Berg, M.G., Younis, I., Kasim, M., Singh, L.N., Wan, L., and Dreyfuss, G. (2010). U1 snRNP protects pre-mRNAs from premature cleavage and polyadenylation. Nature *468*, 664–668.
8. Sperling, J., Azubel, M., and Sperling, R. (2008). Structure and function of the pre-mRNA splicing machine. Structure *16*, 1605–1615.
9. Berg, M.G., Singh, L.N., Younis, I., Liu, Q., Pinto, A.M., Kaida, D., Zhang, Z., Cho, S., Sherrill-Mix, S., Wan, L., and Dreyfuss, G. (2012). U1 snRNP determines mRNA length and regulates isoform expression. Cell *150*, 53–64.
10. Krawczak, M., Thomas, N.S., Hundrieser, B., Mort, M., Wittig, M., Hampe, J., and Cooper, D.N. (2007). Single base-pair substitutions in exon-intron junctions of human genes: nature, distribution, and consequences for mRNA splicing. Hum. Mutat. *28*, 150–158.
11. Baralle, D., and Baralle, M. (2005). Splicing in action: assessing disease causing sequence changes. J. Med. Genet. *42*, 737–748.
12. Fernandez Alanis, E., Pinotti, M., Dal Mas, A., Balestra, D., Cavallari, N., Rogalska, M.E., Bernardi, F., and Pagani, F. (2012). An exon-specific U1 small nuclear RNA (snRNA) strategy to correct splicing defects. Hum. Mol. Genet. *21*, 2389–2398.
13. Pinotti, M., Rizzotto, L., Balestra, D., Lewandowska, M.A., Cavallari, N., Marchetti, G., Bernardi, F., and Pagani, F. (2008). U1-snRNA-mediated rescue of mRNA processing in severe factor VII deficiency. Blood *111*, 2681–2684.
14. Sánchez-Alcudia, R., Pérez, B., Pérez-Cerdá, C., Ugarte, M., and Desviat, L.R. (2011). Overexpression of adapted U1snRNA in patients' cells to correct a 5′ splice site mutation in propionic acidemia. Mol. Genet. Metab. *102*, 134–138.
15. Schmid, F., Glaus, E., Barthelmes, D., Fliegauf, M., Gaspar, H., Nürnberg, G., Nürnberg, P., Omran, H., Berger, W., and Neidhardt, J. (2011). U1 snRNA-mediated gene therapeutic correction of splice defects caused by an exceptionally mild BBS mutation. Hum. Mutat. *32*, 815–824.
16. Schmid, F., Hiller, T., Korner, G., Glaus, E., Berger, W., and Neidhardt, J. (2013). A gene therapeutic approach to correct splice defects with modified U1 and U6 snRNPs. Hum. Gene Ther. *24*, 97–104.
17. Lorson, C.L., Rindt, H., and Shababi, M. (2010). Spinal muscular atrophy: mechanisms and therapeutic strategies. Hum. Mol. Genet. *19* (R1), R111–R118.
18. Hua, Y., Sahashi, K., Rigo, F., Hung, G., Horev, G., Bennett, C.F., and Krainer, A.R. (2011). Peripheral SMN restoration is essential for long-term rescue of a severe spinal muscular atrophy mouse model. Nature *478*, 123–126.

19. Lotti, F., Imlach, W.L., Saieva, L., Beck, E.S., Hao, T., Li, D.K., Jiao, W., Mentis, G.Z., Beattie, C.E., McCabe, B.D., and Pellizzoni, L. (2012). An SMN-dependent U12 splicing event essential for motor circuit function. Cell *151*, 440–454.
20. Imlach, W.L., Beck, E.S., Choi, B.J., Lotti, F., Pellizzoni, L., and McCabe, B.D. (2012). SMN is required for sensory-motor circuit function in *Drosophila*. Cell *151*, 427–439.
21. Fallini, C., Bassell, G.J., and Rossoll, W. (2012). Spinal muscular atrophy: the role of SMN in axonal mRNA regulation. Brain Res. *1462*, 81–92.
22. Schrank, B., Götz, R., Gunnersen, J.M., Ure, J.M., Toyka, K.V., Smith, A.G., and Sendtner, M. (1997). Inactivation of the survival motor neuron gene, a candidate gene for human spinal muscular atrophy, leads to massive cell death in early mouse embryos. Proc. Natl. Acad. Sci. USA *94*, 9920–9925.
23. Paushkin, S., Gubitz, A.K., Massenet, S., and Dreyfuss, G. (2002). The SMN complex, an assemblyosome of ribonucleoproteins. Curr. Opin. Cell Biol. *14*, 305–312.
24. Lefebvre, S., Bürglen, L., Reboullet, S., Clermont, O., Burlet, P., Viollet, L., Benichou, B., Cruaud, C., Millasseau, P., Zeviani, M., et al. (1995). Identification and characterization of a spinal muscular atrophy-determining gene. Cell *80*, 155–165.
25. Lorson, C.L., Hahnen, E., Androphy, E.J., and Wirth, B. (1999). A single nucleotide in the SMN gene regulates splicing and is responsible for spinal muscular atrophy. Proc. Natl. Acad. Sci. USA *96*, 6307–6311.
26. Cartegni, L., and Krainer, A.R. (2002). Disruption of an SF2/ASF-dependent exonic splicing enhancer in SMN2 causes spinal muscular atrophy in the absence of SMN1. Nat. Genet. *30*, 377–384.
27. Kashima, T., and Manley, J.L. (2003). A negative element in SMN2 exon 7 inhibits splicing in spinal muscular atrophy. Nat. Genet. *34*, 460–463.
28. Cho, S., and Dreyfuss, G. (2010). A degron created by SMN2 exon 7 skipping is a principal contributor to spinal muscular atrophy severity. Genes Dev. *24*, 438–442.
29. Hammond, S.M., and Wood, M.J. (2011). Genetic therapies for RNA mis-splicing diseases. Trends Genet. *27*, 196–205.
30. Naryshkin, N.A., Weetall, M., Dakka, A., Narasimhan, J., Zhao, X., Feng, Z., Ling, K.K., Karp, G.M., Qi, H., Woll, M.G., et al. (2014). Motor neuron disease. SMN2 splicing modifiers improve motor function and longevity in mice with spinal muscular atrophy. Science *345*, 688–693.
31. Hua, Y., Vickers, T.A., Okunola, H.L., Bennett, C.F., and Krainer, A.R. (2008). Antisense masking of an hnRNP A1/A2 intronic splicing silencer corrects SMN2 splicing in transgenic mice. Am. J. Hum. Genet. *82*, 834–848.
32. Vezain, M., Saugier-Veber, P., Goina, E., Touraine, R., Manel, V., Toutain, A., Fehrenbach, S., Frébourg, T., Pagani, F., Tosi, M., and Martins, A. (2010). A rare SMN2 variant in a previously unrecognized composite splicing regulatory element induces exon 7 inclusion and reduces the clinical severity of spinal muscular atrophy. Hum. Mutat. *31*, E1110–E1125.
33. Sumner, C.J. (2006). Therapeutics development for spinal muscular atrophy. NeuroRx *3*, 235–245.
34. Blázquez, L., and Fortes, P. (2013). U1 snRNP control of 3′-end processing and the therapeutic application of U1 inhibition combined with RNA interference. Curr. Mol. Med. *13*, 1203–1216.
35. Roca, X., Akerman, M., Gaus, H., Berdeja, A., Bennett, C.F., and Krainer, A.R. (2012). Widespread recognition of 5′ splice sites by noncanonical base-pairing to U1 snRNA involving bulged nucleotides. Genes Dev. *26*, 1098–1109.
36. Furger, A., O'Sullivan, J.M., Binnie, A., Lee, B.A., and Proudfoot, N.J. (2002). Promoter proximal splice sites enhance transcription. Genes Dev. *16*, 2792–2799.
37. Alexander, M.R., Wheatley, A.K., Center, R.J., and Purcell, D.F. (2010). Efficient transcription through an intron requires the binding of an Sm-type U1 snRNP with intact stem loop II to the splice donor. Nucleic Acids Res. *38*, 3041–3053.
38. Le Hir, M., Goyenvalle, A., Peccate, C., Précigout, G., Davies, K.E., Voit, T., Garcia, L., and Lorain, S. (2013). AAV genome loss from dystrophic mouse muscles during AAV-U7 snRNA-mediated exon-skipping therapy. Mol. Ther. *21*, 1551–1558.
39. Hua, Y., Vickers, T.A., Baker, B.F., Bennett, C.F., and Krainer, A.R. (2007). Enhancement of SMN2 exon 7 inclusion by antisense oligonucleotides targeting the exon. PLoS Biol. *5*, e73.
40. Marquis, J., Meyer, K., Angehrn, L., Kämpfer, S.S., Rothen-Rutishauser, B., and Schümperli, D. (2007). Spinal muscular atrophy: SMN2 pre-mRNA splicing corrected by a U7 snRNA derivative carrying a splicing enhancer sequence. Mol. Ther. *15*, 1479–1486.
41. Skordis, L.A., Dunckley, M.G., Yue, B., Eperon, I.C., and Muntoni, F. (2003). Bifunctional antisense oligonucleotides provide a trans-acting splicing enhancer that stimulates SMN2 gene expression in patient fibroblasts. Proc. Natl. Acad. Sci. USA *100*, 4114–4119.
42. Madocsai, C., Lim, S.R., Geib, T., Lam, B.J., and Hertel, K.J. (2005). Correction of SMN2 pre-mRNA splicing by antisense U7 small nuclear RNAs. Mol. Ther. *12*, 1013–1022.
43. Kwek, K.Y., Murphy, S., Furger, A., Thomas, B., O'Gorman, W., Kimura, H., Proudfoot, N.J., and Akoulitchev, A. (2002). U1 snRNA associates with TFIIH and regulates transcriptional initiation. Nat. Struct. Biol. *9*, 800–805.
44. Le, T.T., Pham, L.T., Butchbach, M.E., Zhang, H.L., Monani, U.R., Coovert, D.D., Gavrilina, T.O., Xing, L., Bassell, G.J., and Burghes, A.H. (2005). SMNDelta7, the major product of the centromeric survival motor neuron (SMN2) gene, extends survival in mice with spinal muscular atrophy and associates with full-length SMN. Hum. Mol. Genet. *14*, 845–857.
45. Wang, J., and Dreyfuss, G. (2001). Characterization of functional domains of the SMN protein in vivo. J. Biol. Chem. *276*, 45387–45393.
46. Scholl, R., Marquis, J., Meyer, K., and Schümperli, D. (2007). Spinal muscular atrophy: position and functional importance of the branch site preceding SMN exon 7. RNA Biol. *4*, 34–37.
47. Cavallari, N., Balestra, D., Branchini, A., Maestri, I., Chuamsunrit, A., Sasanakul, W., Mariani, G., Pagani, F., Bernardi, F., and Pinotti, M. (2012). Activation of a cryptic splice site in a potentially lethal coagulation defect accounts for a functional protein variant. Biochim. Biophys. Acta *1822*, 1109–1113.
48. Monani, U.R., Coovert, D.D., and Burghes, A.H. (2000). Animal models of spinal muscular atrophy. Hum. Mol. Genet. *9*, 2451–2457.

The American Journal of Human Genetics
Supplemental Data

Improvement of SMN2 Pre-mRNA Processing Mediated by Exon-Specific U1 Small Nuclear RNA

Andrea Dal Mas, Malgorzata Ewa Rogalska, Erica Bussani, and Franco Pagani

Supplementary Material

S1

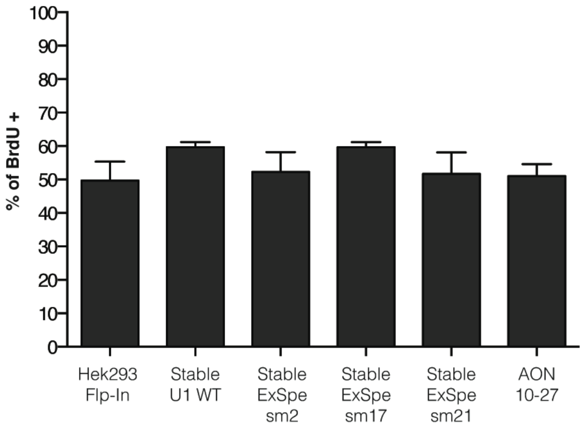

Figure S1.

Proliferation assay for Hek293 Flp-In cells, ExSpe U1 or U1 WT stable clones. BrdU staining of cells expressing one additional copy of U1 snRNA (U1 WT) gene ore one copy of ExSpe U1 sm2, sm 17 or sm21 genes. The histograms represent the percentage of BrdU positive cells (BrdU+) and are the mean +/- SD of three independent experiments.

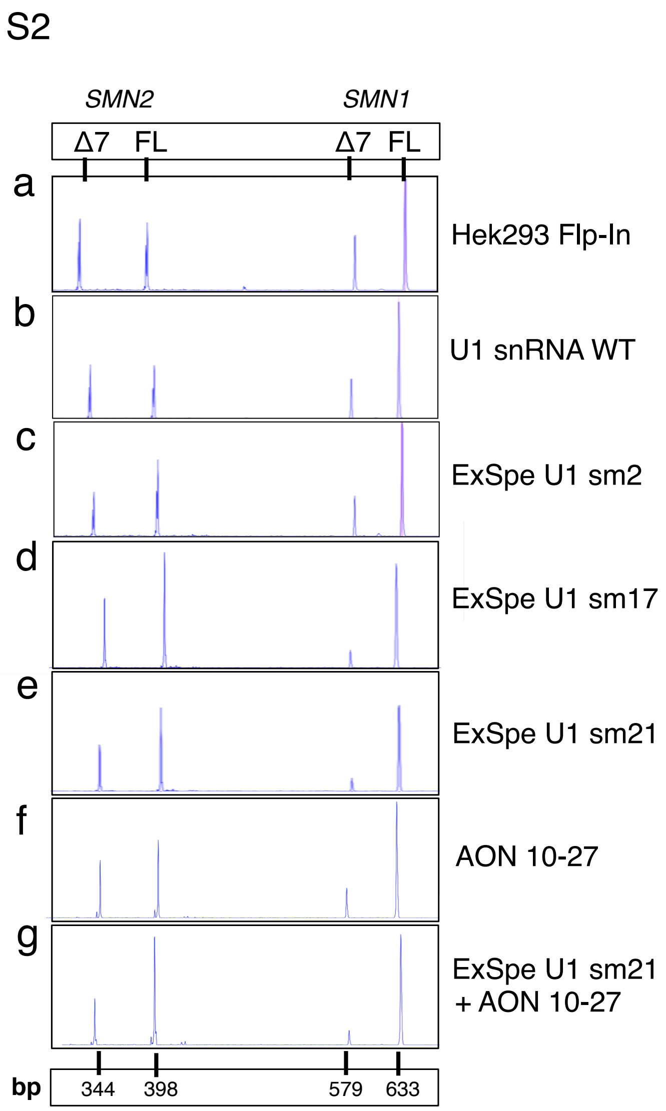

Figure S2.

Capillary electrophoresis output of SMN1 and SMN2 transcripts in Hek293 Flp-In cells. Total RNA was extracted from Hek293 Flp-In cells stably expressing one copy of U1 WT or ExSpe sm2, sm17 or sm21 and cells transfected with antisense oligonucleotide 10-27 (AON 10-27) and ExSpe sm21 stable clone transfected with AON 10-27. RT-PCR was performed with FAM-labelled-E6-Fw and E8-467-Rev primers. The corresponding amplified products were digested with DdeI restriction enzyme in order to discriminate fragments coming from *SMN1* or *SMN2.* The resulting products were run on capillary electrophoresis. Each peak reported in the graph represent an isoform and the area surrounding the peaks gives the relative quantification of the corresponding isoforms. The identity of each peak (FL containing exon 7 or ΔE7 with exon 7 skipped) and its provenience (*SMN1 or SMN2*) is indicated on the top of the panel. The size is indicated below.

S3

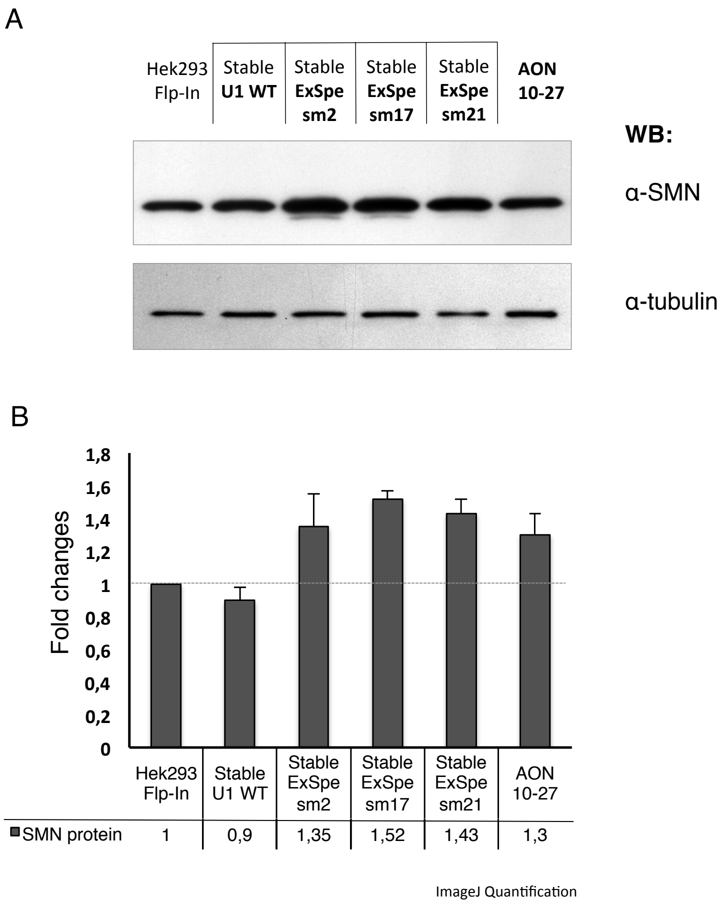

Figure S3

Expression of ExSpe U1 from a single chromosome-integrated copy of the gene increases the amount of SMN protein in Hek293 Flp-In cells.

(a) Protein extract from Hek293 Flp-In stable clones expressing one copy of ExSpe U1 sm2, sm17, sm21 or U1 WT were loaded on 4-12% SDS-PAGE. SMN protein was detected by Western blotting and the same blot was re-probed with antibody against tubulin as a protein loading control.

(b) Intensity of protein bands in (a) was estimated through ImageJ-Quantification program. The histograms indicate the SMN protein fold increase relative to the amount of SMN protein in Hek293 Flp-In (value set to 1). Error bars show standard deviation.

S4

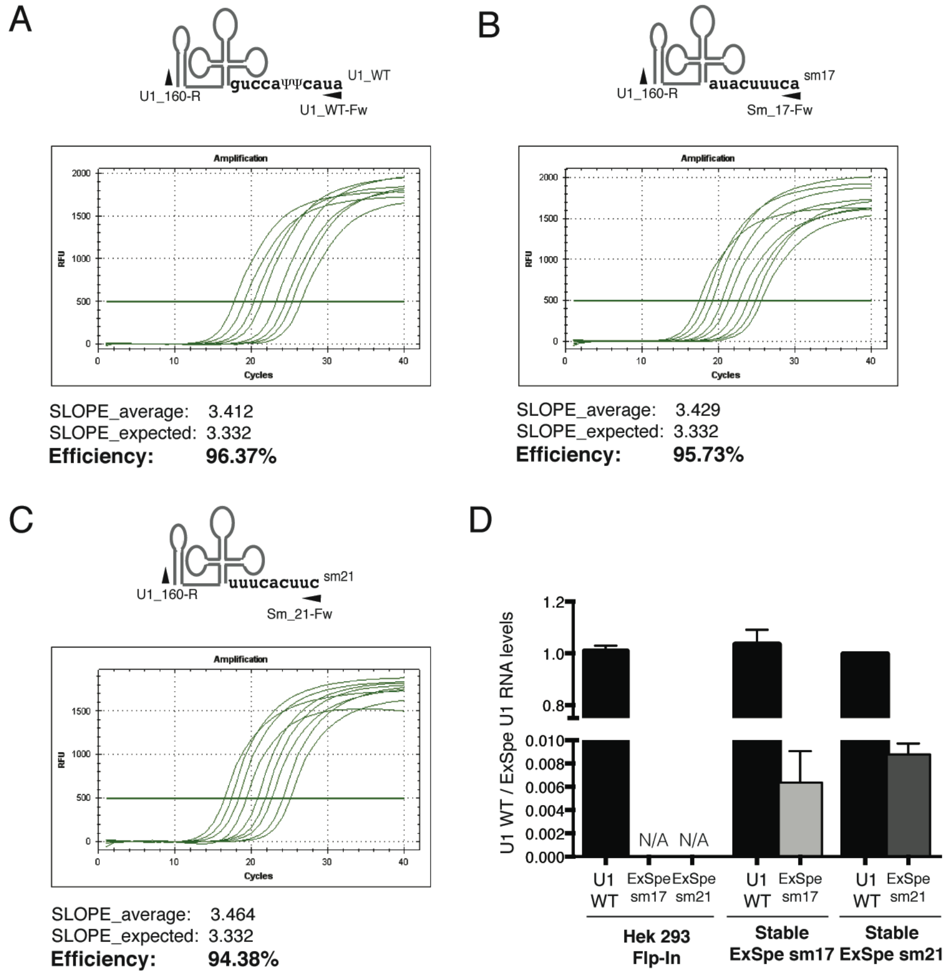

Figure S4.

Analysis of ExSpe U1 sm17 and sm21 expression levels.

(a-b-c) The three RT-PCR reactions used to determine the levels of U1 snRNA WT, ExSpe sm17 and ExSpe sm21 have equivalent efficiencies. ExSpe U1 sm2 was not analysed, as PCR could not discriminate between ExSpe U1 sm2 and U1 snRNA 5'-tails, due to the high sequence similarity. Plasmid DNA dilutions were used as template to determine the efficiencies of PCR reactions. Calibration curves show that the efficiencies are similar, thus allowing to compare them directly and to estimate absolute levels of ExSpe U1s.

(d) Total RNA was extracted from the indicated cells and treated with DNAse. Retrotranscription reaction has been performed +/-RT to check for DNA contamination. Sybr-based qPCR has been performed using specific primers to detect U1 snRNA WT, ExSpe U1 sm17 and ExSpe U1 sm21. The amount of ExSpe U1 for each cell line has been expressed as ratio to endogenous U1 snRNA WT level.

S5

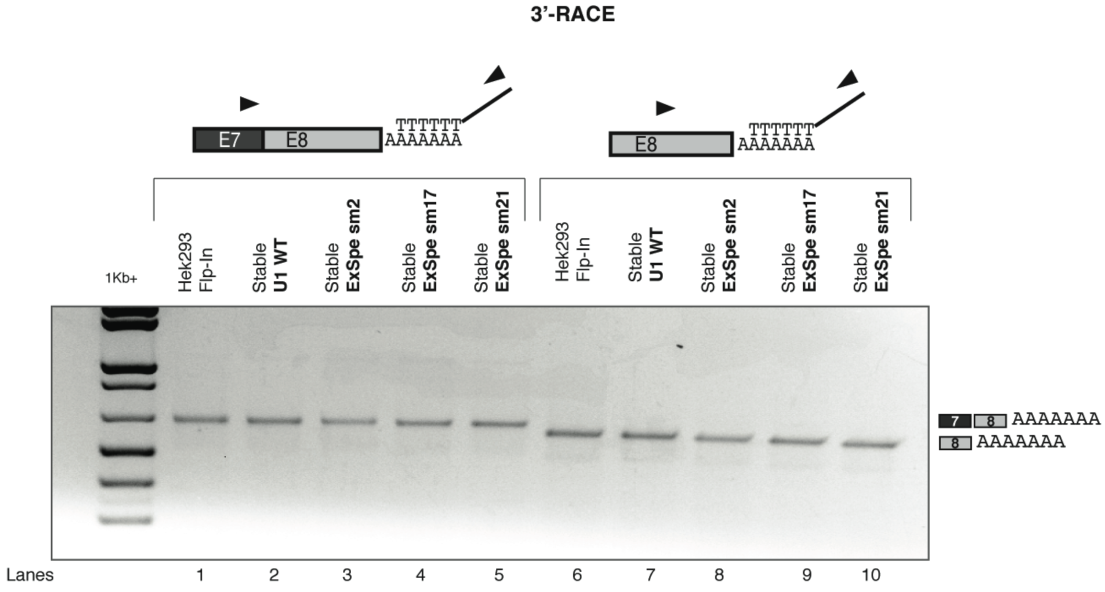

Figure S5.

3'-RACE (Rapid amplification of cDNA ends) assay. Total RNA was extracted from the indicated cells and retrotranscribed using an oligo dT primer with a specific anchor. Polyadenylated transcripts from SMN genes were evaluated using a primer located on exon 7 (lanes 1-5) or on exon 8 (lanes 6-10) in combination with a specific primer located on the anchor. The amplified fragments were separated on 1.5% agarose gel. The identity of the bands was verified through direct sequencing of the PCR products and has been schematically reported on the right of the gel.

S6

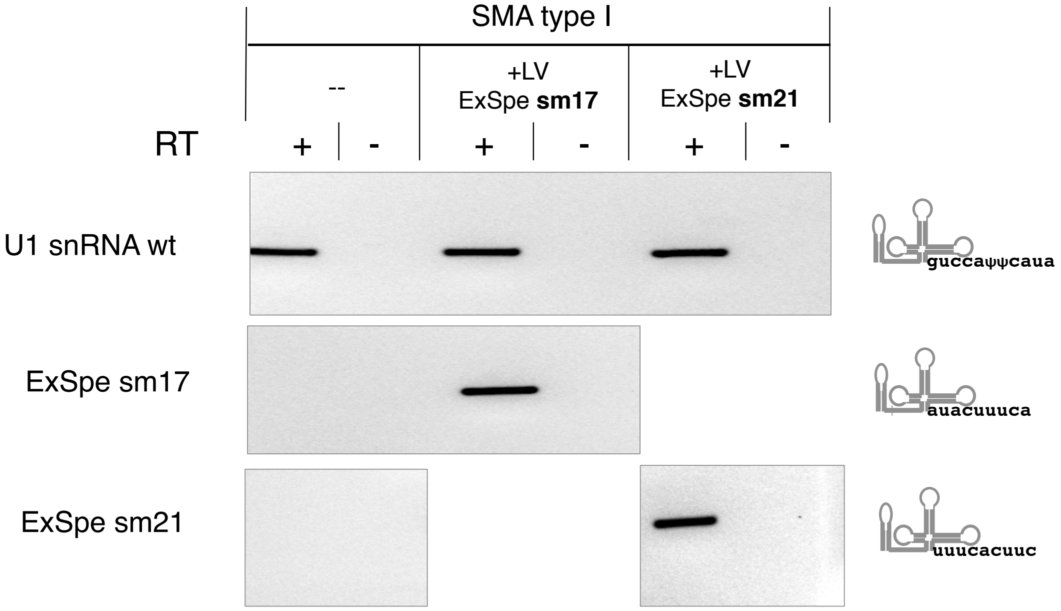

Figure S6.

Expression of Exon-Specific U1 RNAs after lentiviral transduction in SMA type I fibroblasts. Total RNA was extracted from SMA type I fibroblasts treated with lentiviral particle expressing ExSpe U1 sm17 or sm21 and treated with DNAse. RT-PCR has been performed +/- retrotranscriptase (+/-RT) to check for DNA contamination using specific set of primers to amplify the endogenous U1 WT, the ExSpe sm17 or the ExSpe sm21. The primers used for each amplification are indicated on the left of the panel and the identity of the PCR product is schematically reproduced on the right.

Primer name	Sequence (5'- -3')
E6-Fw_FAM	5'-*FAM*-ATAATTCCCCCACCACCTCCC-3'
E8-467-Rev	5'-TTGCCACATACGCCTCACATAC-3'
U1sm17-Fw	5'-ATAACTTTCATAGCAGGGGGAGAT-3'
U1sm21-Fw	5'-ATAATTCACTTTGCAGGGGGAGAT-3'
U1-160 Rev	5'-GGAAAGCGCGAACGCAGTC-3'
Ex2a Fw	5'-AGCGATGATTCTGACATTTGGGATG-3'
Ex2b Rev	5'-CTGTTGTAAGGAAGCTGCAGTATTCTT-3'
Int1_13850Fw	5'-GATTAAACCTATCTGAACATGAG-3'
Int2a_14006 Rev	5'-GAAATAAGAAAACGACTAAGCAAG-3'
Int6 Fw	5'-CCATATAAGCTATCTATATATAGCTATCT-3'
Int7 Rev	5'-CATTTGTTTTCCACAAACCATAAAGTT -3'
18S Fw	5'-CCTTTAACGAGGATCCATTGGA -3'
18S Rev	5'-CGAGCTTTTTAACTGCAGCAACT-3'
Oligo dT for 3'-RACE	5'-GACCACGCGTATCGATGTCGACTTTTTTTTTTTTTTTTV-3'
Exon-6_Fw	5'-TGCTTTGGGAAGTATGTTAATTTCATG-3'
Exon-7_Fw	5'-GTGCTCACATTCCTTAAATTAAGG-3'
Exon-8_Fw	5'-GGCATAGAGCAGCACTAAATGACACC-3'
Anchor Rev	5'-GACCACGCGTATCGATGTCGAC-3'

Table S1.

Sequences of the DNA oligonucleotides used for RT-PCR experiments.
